# Compact Tabletop Magnetic Resonance Elastography for Mapping Soft Tissue Viscoelasticity

**DOI:** 10.1002/advs.75728

**Published:** 2026-05-19

**Authors:** Weijie Zhao, Lisa‐Marie Skrip, Heiko Tzschätzsch, Tom Meyer, Hossein S. Aghamiry, Alexander Arnold, Lene Änne Böhne, Jakob Jordan, Oliver Boehm, Agnes K. Boehm, Steffen Görner, Jakob Schattenfroh, Yanglei Wu, Pedro Augusto Dantas de Moraes, Johann Pratschke, Jürgen Braun, Igor M. Sauer, Jing Guo, Ingolf Sack, Karl H. Hillebrandt

**Affiliations:** ^1^ Department of Radiology Charité – Universitätsmedizin Berlin Corporate Member of Freie Universität Berlin and Humboldt‐Universität zu Berlin Berlin Germany; ^2^ Department of Surgery Experimental Surgery, CCM|CVK Charité – Universitätsmedizin Berlin Corporate Member of Freie Universität Berlin and Humboldt‐Universität zu Berlin Berlin Germany; ^3^ Institute of Medical Informatics Charité – Universitätsmedizin Berlin Corporate Member of Freie Universität Berlin and Humboldt‐Universität zu Berlin Berlin Germany; ^4^ Institute of Pathology Charité – Universitätsmedizin Berlin Corporate Member of Freie Universität Berlin and Humboldt‐Universität zu Berlin Berlin Germany; ^5^ BIH Charité Digital Clinician Scientist Program Berlin Institute of Health at Charité – Universitätsmedizin Berlin BIH Biomedical Innovation Academy Berlin Germany

**Keywords:** chemotherapy treatment response, colorectal liver metastasis, intratumoral biomechanical heterogeneity, magnetic resonance elastography, tabletop magnetic resonance elastography

## Abstract

Compact, cost‐effective tabletop magnetic resonance elastography (ttMRE) is an emerging research tool for assessing bulk soft tissue biomechanics but still lacks spatially resolved imaging of viscoelastic properties in heterogeneous specimens like tumors. Here, we develop and validate ttMRE for quantifying intratumoral biomechanical heterogeneity in human colorectal liver metastasis (CRLM). We excite cylindrical, multifrequency shear waves and reconstruct viscoelastic maps using adaptive bandpass filters constrained by rheological Bessel‐fit priors. In this way, we obtain biophysical markers that agree with ground‐truth measurements in numerical and viscoelastic phantoms. In CRLM, shear‐wave‐speed and penetration‐rate maps – proxies of stiffness and attenuation, respectively – as well as their derived interquartile range and Shannon entropy, suggest markedly less viscous, stiffer, and more biomechanical heterogeneous tissue in specimens from responders to chemotherapy versus non‐responders, with heterogeneity metrics outperforming direct viscoelastic parameters in diagnostic accuracy. These findings establish compact, automated ttMRE as a low‐cost, scalable platform for spatial biomechanical profiling and quantitative assessment of treatment response in bulky colorectal liver metastasis specimens.

## Graphical abstract

This work introduces a compact, low‐cost tabletop magnetic resonance elastography platform for high‐resolution viscoelastic mapping in soft‐tissue specimens. Using this method in human colorectal liver metastases, we demonstrate fully automated biomechanical profiling of treatment response and show that heterogeneity‐based metrics outperform conventional viscoelastic averages in distinguishing chemotherapy responders from non‐responders.

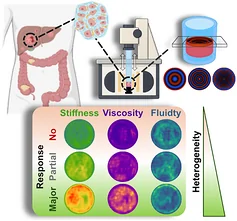

## 1 Introduction

Tumor heterogeneity is a crucial determinant of cancer progression, treatment response, and patient survival [1]. The biomechanical hallmarks of solid tumors reflect cancer cell viability, allowing them to be assessed for tumor staging and treatment monitoring [2, 3, 4]. Magnetic resonance elastography (MRE) is a unique imaging method for noninvasively mapping viscoelastic properties of tumors in vivo [5, 6]. When used in combination with multiparametric magnetic resonance imaging (MRI), MRE adds diagnostic value to the detection and imaging‐based characterization of solid tumors in vivo, including those of the prostate, colon, and liver [7, 8, 9]. However, despite clinical and technical advances, in vivo MRE remains limited in spatial resolution often with voxel sizes > 2 × 2 × 2 mm^3^ and detection limits >10 mm lesion diameter [6, 9, 10] – which often hampers the analysis of intratumoral biomechanical heterogeneity [6].

**FIGURE 1:**
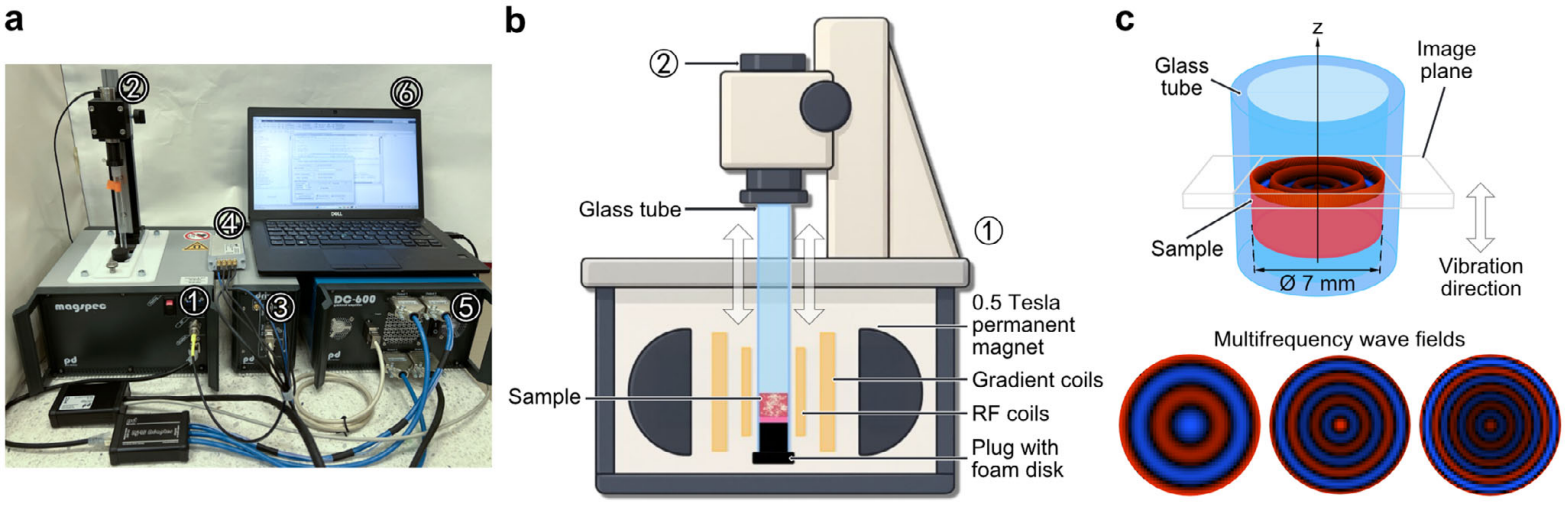
Setup of benchtop MRE for multifrequency viscoelasticity mapping. (a) Compact ttMRE setup comprising (1) 0.5‐Tesla MRI scanner, (2) piezoelectric actuator, (3) console, (4) signal preamplifier, (5) gradient amplifier, and (6) control laptop with MATLAB‐based interface. (b) Diagram of sample orientation inside the MRI scanner. Specimens are embedded in a cylindrical glass tube and placed within a permanent magnet equipped with radio frequency (RF) and gradient coils. Harmonic mechanical vibrations are generated by a piezoelectric actuator mounted on the upper end of the tube. The main vibration direction is along the cylinder axis (arrows). Shear wave propagation through the sample is encoded using a spin‐echo‐MR sequence with motion‐encoding gradients synchronized to the mechanical vibrations [24]. (c) Sample geometry within the glass tube, image plane, vibration direction, and acquired wave fields. Representative simulated wave fields with damping at 1200, 2000, and 3000 Hz, illustrating cylindrical wave propagation and frequency‐dependent attenuation within the sample volume.

Complementary to clinical MRE, Bessel‐function‐based cylindrical wave field processing methods enable automated multifrequency mechanical testing of small cylindrical tissue samples [11, 12]. In particular, compact low‐field tabletop MRE (ttMRE) has emerged as a cost‐effective research platform for investigating small cylindrical tissue samples of approximately 3.5 mm radius, albeit without spatially resolved measurements across the sample [4, 13, 14, 15, 16, 17, 18, 19]. In a number of different studies, ttMRE has been used to distinguish cell viability and treatment response of human colorectal liver metastases (CRLM) [20], to assess cell motility in a human prostate cancer model [18], and to study fluid‐solid interactions in pancreatic extracellular matrix scaffolds [17]. Unlike rheometry or indentation‐based force‐feedback measurement, ttMRE characterizes bulky tissue properties independent of surface texture and probe contact and can be easily combined with other MRI techniques such as water diffusion or fat fraction quantification [19]. Therefore, ttMRE has emerged as a reference and ground‐truth modality for many MRE studies in more complex tissue geometries [21, 22] and has even been adapted to ultrasound [23]. The setup, shown in Figure 1, induces and acquires cylindrical wave displacement parallel to the main cylinder axis over a frequency range from 200 to 6000 Hz [17, 24] and reconstructs three biomechanical parameters: shear wave speed *c* (how fast waves propagate, a proxy of stiffness), shear wave penetration rate *a* (how deep waves penetrate at a specific frequency, a proxy of inverse attenuation or viscous loss), and loss angle *φ* (the elastic–viscous ratio, ranging from 0 for a pure elastic solid to π/2 for a viscous fluid) [5, 24]. Together, these parameters provide a comprehensive picture of global tissue viscoelasticity; however, without allowing generation of spatially resolved maps.

To translate ttMRE into a clinical diagnostic modality, we address the lack of spatial resolution by introducing a multifrequency wave inversion framework. This framework yields spatially resolved viscoelasticity maps of bulky soft tissue specimens at low cost and with submillimeter pixel resolution, and can be readily integrated into multiparametric MRI protocols. The feasibility of the fully automated postprocessing pipeline is validated in wave simulations to estimate the resolution limits, viscoelastic phantoms to confirm the quantitative agreement with reference measurements, and resected human CRLM specimens to stratify responses to chemotherapy. Mechanical profiling of liver metastases at surgery provides objective information on cell viability and treatment efficacy and has the potential to inform clinical decision‐making [20].

## 2 Results

### 2.1 Wave Simulations

**FIGURE 2:**
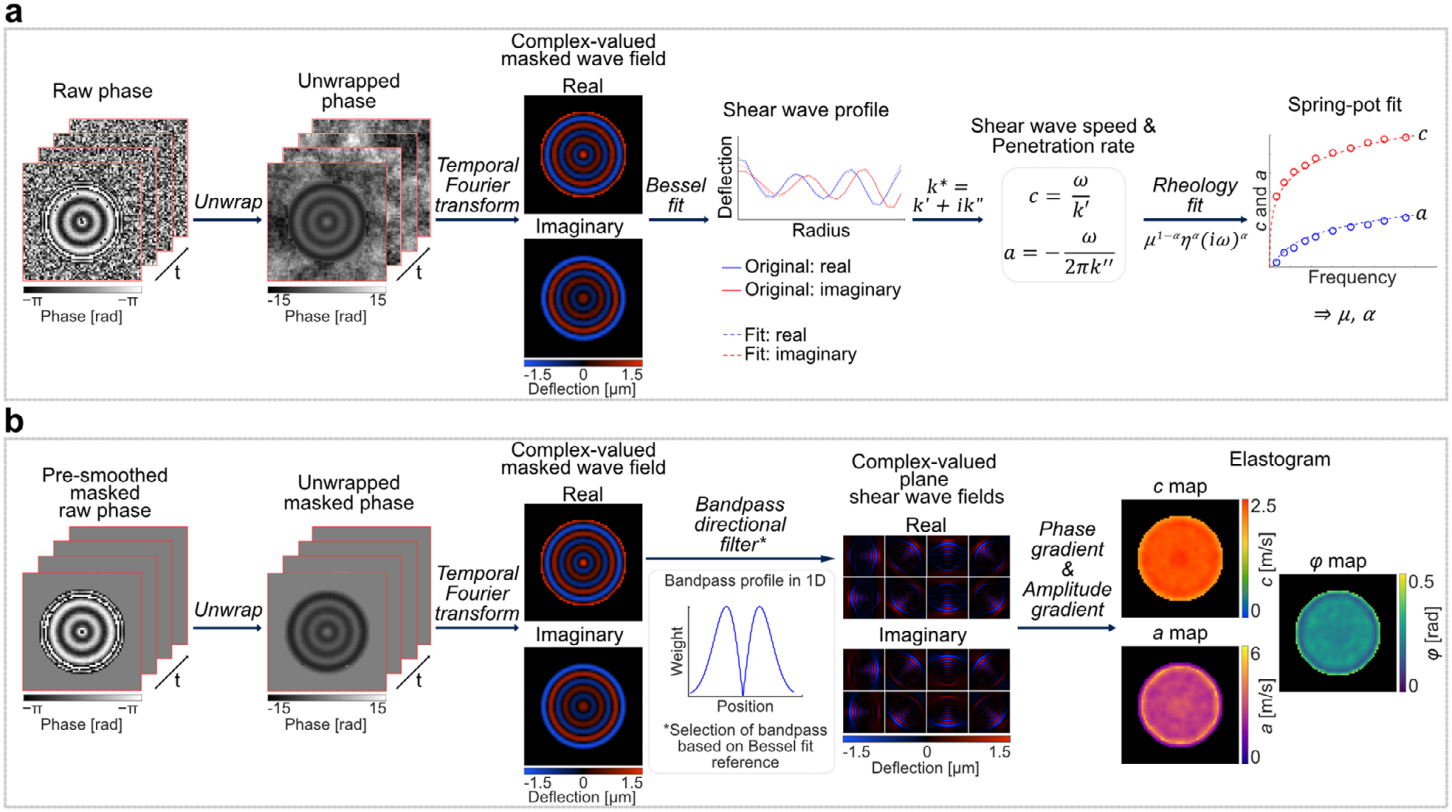
Comparison of Bessel‐fit and spatially resolved postprocessing pipeline for multifrequency ttMRE data. (a) A phase‐unwrapping algorithm was applied to the raw MRE data, followed by a temporal Fourier transform to obtain a complex‐valued wave field at fundamental drive frequency. A Bessel function of the first kind [24] was fit to the masked wave field to derive a shear wave profile, assuming cylindrical wave propagation in a mechanically homogenous medium. The resulting complex‐valued wavenumber *k* ^*^ was used to calculate global bulk‐averaged shear wave speed *c* and penetration rate *a*. Rheological parameters were subsequently obtained by fitting *c* and *a* across multiple frequencies using rheological models such as the spring‐pot [26]. (b) To generate contrast, a spatially resolved multifrequency inversion pipeline was developed to capture the mechanical heterogeneity and avoid assumptions about global tissue homogeneity. Therefore, the raw MRE data was first smoothed and masked prior to unwrapping and temporal Fourier transform. Then, the complex‐valued wave field was filtered using bandpass directional filters to extract unidirectional plane shear wave components. The thresholds of the bandpass filters were automatically adapted based on the Bessel fit reference shown in (a), which served as priors to frequency‐specific bandpass in the individual sample. These filtered plane wave fields were then used to compute spatial gradients of phase and amplitude images, which were compounded across directions and frequencies for spatially resolved parameter maps of shear wave speed *c* and penetration rate *a* according to Equations 7 and 8. Loss angle *φ* was derived from *c* and *a*. Note, only one frequency is depicted in the figure for clarity, while both processing pipelines were independently applied to multifrequency ttMRE data.

Conventional ttMRE benefits from the cylindrical geometry of tissue samples, which favors analytical solutions of the ill‐posed inverse problem in MRE but prevents mapping [24]. To combine the best of two worlds, we developed adaptive bandpass filters for multifrequency inversion [25] which were automatically selected by prior Bessel fits of shear wave speed (*c*) and penetration rate (*a*) over excitation frequency, as illustrated in Figure 2. *c* and *a* were obtained from previous Bessel‐fit wave images as part of the standard ttMRE pipeline [24].

**FIGURE 3:**
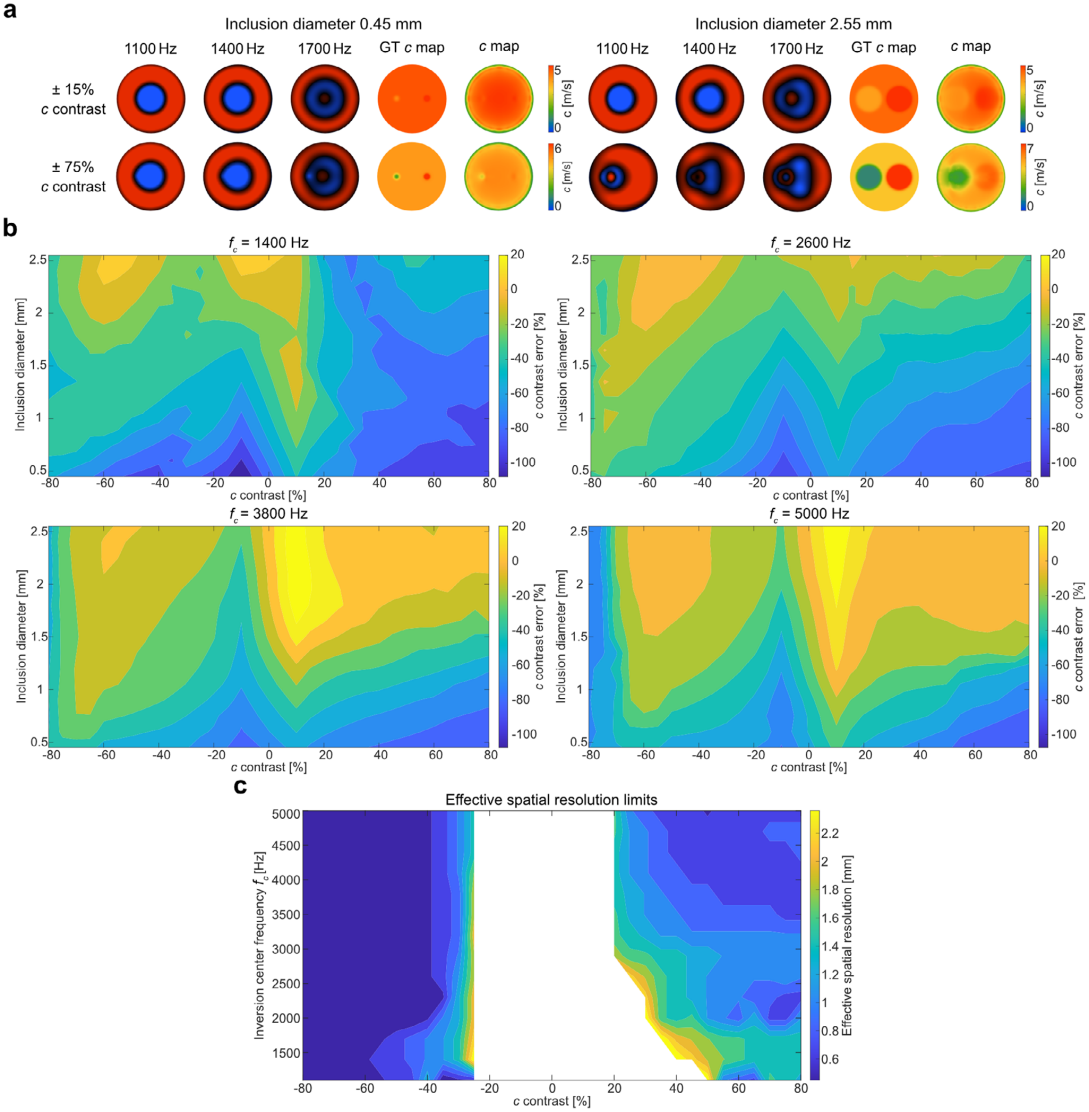
Wave simulation results for estimating the effective resolution limits of the spatially resolved postprocessing pipeline for detecting small inclusions. (a) The simulation results for two different inclusion sizes (0.45 mm, left panel; 2.55 mm, right panel) with shear wave speed *c* contrasts of ±15% (top row) and ±75% (bottom row). The first three columns of each panel show the real part of the wave fields at three representative frequencies (color bar omitted; values correspond to displacement). The fourth column shows the ground truth (GT) *c* maps averaged across three frequencies, and the fifth column shows the reconstructed *c* maps obtained using the multifrequency spatially resolved postprocessing pipeline. (b) Contour maps of the reconstructed *c* contrast error as a function of inclusion diameter (0.45–2.55 mm) and GT *c* contrast (−80% to 80%) at four inversion center frequencies (1400, 2600, 3800, and 5000 Hz). The color bar represents the error of the reconstructed *c* contrast error. For practical interpretation, absolute errors in *c* contrast below 10% were considered acceptable [27]. (c) Error contour map for effective spatial resolution limits in relation to GT *c* contrast and center frequency. The color bar indicates the minimum detectable inclusion size. The blank region indicates conditions where inclusions were not detectable according to the defined criteria.

To first estimate the resolution limits, we applied the entire mapping pipeline to simulated data. Figure 3a shows representative wave simulations and corresponding maps. In all cases, three inversion frequencies ranging from 1100 to 1700 Hz with a center frequency of 1400 Hz were used. The real parts of the simulated wave fields are shown, demonstrating local variations in wavelength across spaces and frequencies. Two inclusion sizes are presented: 0.45 mm (left panel) and 2.55 mm (right panel) with ±15% *c* contrast (top row) and ±75% *c* contrast (bottom row). The ground truth (GT) *c* maps averaged over the three frequencies are shown alongside reconstructed *c* maps obtained by our proposed postprocessing pipeline. Overall, larger inclusions and higher contrast levels were reconstructed more accurately. Notably, negative *c* contrasts (softer inclusions) exhibited higher detectability than positive contrasts (stiffer inclusions). These observations are quantitatively displayed in Figure 3b where contour maps of the error between GT and reconstructed *c* contrast versus inclusion size are shown for four center frequencies. At lower frequencies, larger contrast errors occur, particularly for small inclusions. Across all frequencies, softer inclusions (negative *c* contrast) consistently exhibited lower reconstruction errors and thus better effective resolutions than stiffer inclusions (positive *c* contrast). For example, at 1400 Hz, inclusions smaller than 1 mm with a *c* contrast below −40% showed *c* contrast errors below 50%, whereas stiffer inclusions required larger sizes to be detected. At higher frequencies, the reconstruction accuracy further improved for small inclusions. For example, at 3800 Hz, inclusions of only 0.75 mm diameter and ±20% contrast could still be detected with an error <50%. Figure 3c summarizes the effective spatial resolution limits as a function of center frequency and *c* contrast. In the low‐contrast regime between approximately −20% to +20%, no inclusion could be detected, making the definition of resolution limits infeasible. Again, higher excitation frequencies, and stronger *c* contrast improved the detectability of small inclusions, while softer inclusions had superior effective resolutions compared to stiffer ones under otherwise identical conditions.

### 2.2 Phantom Validation

Viscoelastic mapping was further validated in (1) a homogeneous viscoelastic phantom, (2) a heterogeneous elastic phantom, and (3) a heterogeneous viscoelastic phantom.

**FIGURE 4:**
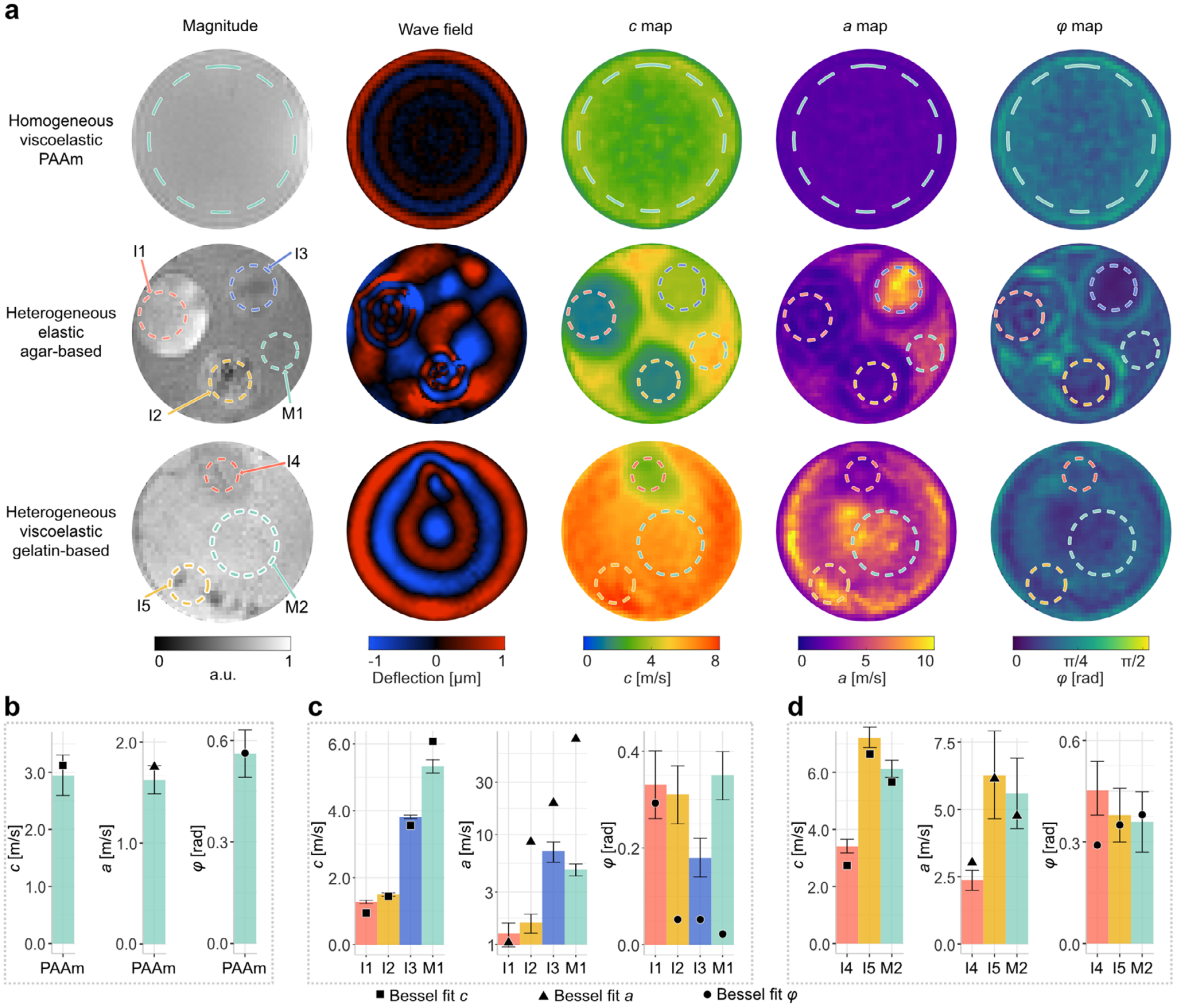
Biomechanical parameter maps and results in three phantoms. (a) ttMRE magnitude image, wave field (real part) at a representative acquisition frequency, and biomechanical parameter maps of shear wave speed *c*, penetration rate *a*, and loss angle *φ* for each phantom. The PAAm [28] phantom was composed of homogeneous viscoelastic material. The heterogeneous elastic agar‐based phantom comprised three inclusions: I1 (ultrasound gel), I2 (0.5 wt.% agar), and I3 (1.2 wt.% agar), embedded within the matrix M1 (1.8 wt.% agar). The heterogeneous viscoelastic gelatin‐based phantom contained two inclusions: I4 (10 wt.% gelatin) and I5 (35 wt.% gelatin), within the matrix M2 (25 wt.% gelatin). Regions of interest (ROIs) for quantitative analysis are indicated by dashed lines with colors corresponding to the bar plots in (b‐d). (b–d) ROI‐based comparison of biomechanical parameter maps with reference values obtained by Bessel fitting of (b) the PAAm phantom itself, and the ground‐truth homogeneous phantoms for the (c) agar‐ and (d) gelatin‐based phantom inclusion materials. Bar plots with error bars show mean ± standard deviation across pixels within each analyzed region, demarcated in the biomechanical parameter maps.

Figure 4a shows representative ttMRE magnitude images, wave images, and biomechanical parameter maps of all three phantoms. The heterogeneity in phantoms (2) and (3) is apparent, demonstrated by the local changes in wavelength and reflected in the parameter maps. The average values within each inclusion and matrix were compared with the reference values obtained by Bessel fitting of homogeneous phantoms made of the same materials as used for the inclusions and matrix of the heterogeneous phantoms. For the homogeneous viscoelastic linear polymerized polyacrylamide (PAAm) [28] phantom (1), the values obtained by ttMRE mapping agreed well with Bessel‐fit reference values, with absolute deviations of <4%, <6%, and <2% for *c*, *a*, and *φ*, respectively (Figure 4b). In the heterogeneous elastic phantom (2), *c* increased linearly with agar concentration, ranging from 1.5 ± 0.1 m/s in 0.5 wt.% agar (inclusion 2) to 3.8 ± 0.1 m/s in 1.2 wt.% agar (inclusion 3), and 5.3 ± 0.3 m/s in the 1.8 wt.% agar matrix. Stiffness differences among the agar gels on the same order were confirmed by Bessel fitting. The ultrasound gel (inclusion 1) showed a *c* value of 1.3 ± 0.1 m/s. Comparison of mapping and Bessel‐fit results revealed consistent trends in *c* across all inclusions and matrix, with a relative difference <29%. However, a disparity of >88% was observed in the viscosity‐related parameters *a* and *φ* within the agar regions, indicating unreliable viscosity quantification in a nearly pure elastic material (Figure 4c). In contrast, the heterogeneous viscoelastic phantom (3) with higher viscosity showed higher agreement between mapping and Bessel‐fit reference values in all inclusions and matrix, with relative differences of <28% for *c*, <21% for *a*, and <49% for the elastic–viscous ratio parameter *φ* (Figure 4d). All biomechanical results obtained in the phantom experiments are compiled in Table S2. Furthermore, the mechanical properties of all materials were independently characterized using shear oscillatory rheometry. The corresponding validation results are provided in Note S1.

Biomechanical heterogeneity was quantified from biomechanical parameter maps using interquartile range (IQR, spread), Shannon entropy (*H*, distributional randomness), and joint metrics combining biomechanical parameters to capture the multidimensional complexity of the distribution. The joint IQR, defined as the product of the IQRs of *c* and *a*, and the joint Shannon entropy, derived from the joint distribution of *c* and *a*, provided enhanced sensitivity than conventional variability measures [29, 30, 31]. As expected, the homogeneous viscoelastic phantom (1) showed the least heterogeneity according to these parameters. Specifically, the joint IQR and Shannon entropy increased from 0.2 ± 0.0 m^2^/s^2^ and 3.1 ± 0.0 bits in the homogeneous phantom (1) to 3.9 ± 0.2 m^2^/s^2^ and 5.9 ± 0.0 bits, and 2.9 ± 0.2 m^2^/s^2^ and 6.3 ± 0.0 bits in agar‐ (2) and gelatin‐based (3) heterogeneous phantoms, respectively. All other heterogeneity metrics increased from phantom (1) to phantoms (2) and (3). Complete biomechanical heterogeneity values are provided in Table S2.

### 2.3 Quantitative Biomechanical Mapping of CRLM Tissue Samples

**FIGURE 5:**
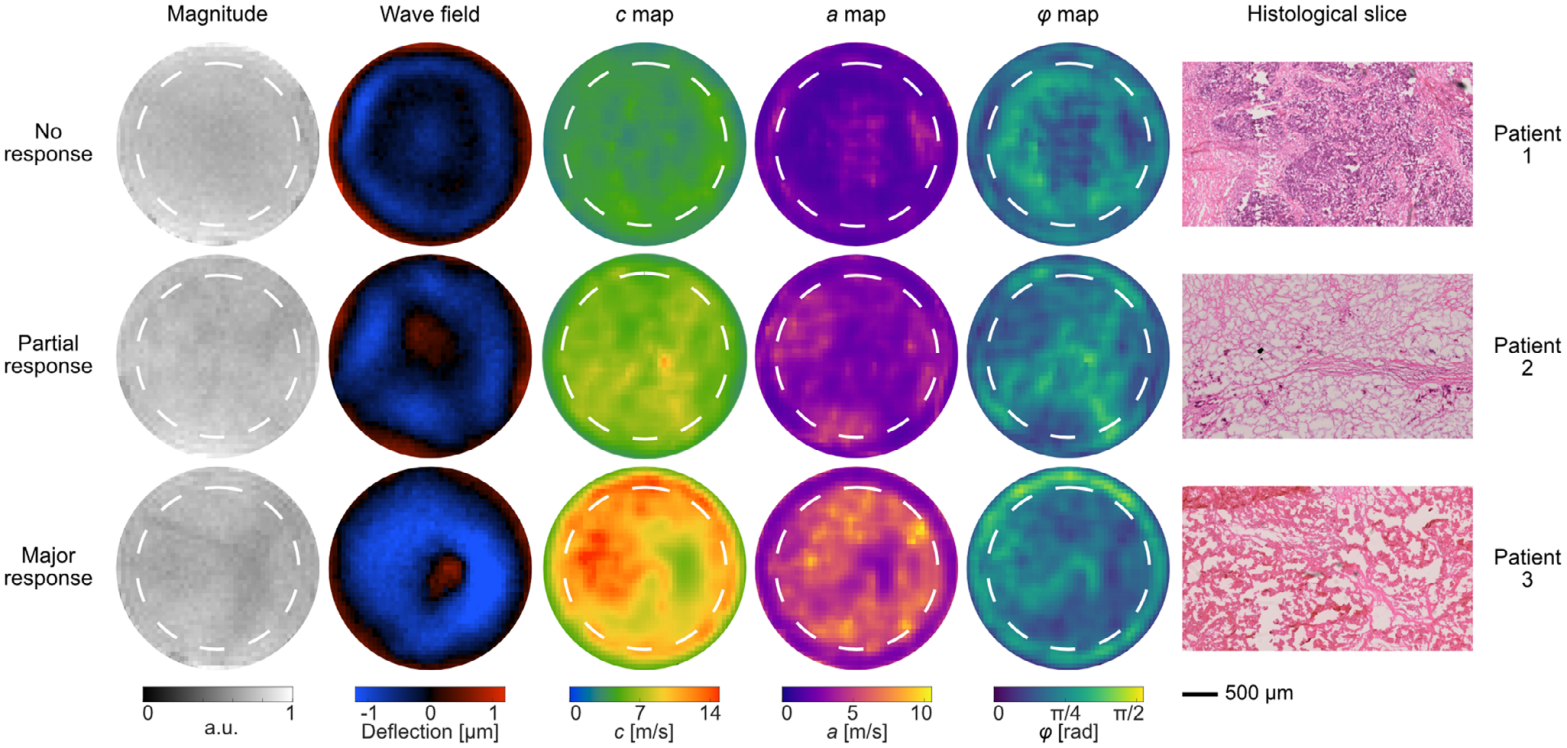
Biomechanical parameter mapping in CRLM tumor specimens. Samples from three patients illustrate distinct treatment responses: no response (patient 1), partial response (patient 2), and major response (patient 3). Each column shows the ttMRE magnitude image, wave field (real part) at a representative acquisition frequency, and biomechanical parameter maps of shear wave speed *c*, penetration rate *a*, and loss angle *φ*, and the corresponding H&E‐stained histological section. Dashed circles indicate the ROI. For histology, a rectangular region corresponding to the measured ttMRE slice is shown.

Thirty‐four human CRLM samples were divided into three groups based on histological grading performed to assess the pathological response to chemotherapy: major response (n = 5), partial response (n = 6), and no response (n = 23). Figure 5 shows ttMRE magnitude images, wave images, and reconstructed biomechanical parameter maps alongside hematoxylin and eosin (H&E) stained histological images from one representative patient of each treatment response group. In these examples, *c* and *a* maps appeared more heterogeneous with higher stiffness and viscosity values in major responders than in partial or non‐responders. The corresponding histological images revealed mixed regions of residual tumor and treatment‐induced fibrosis in partial responders as well as fibrotic stroma and necrotic debris in major responders.

**FIGURE 6:**
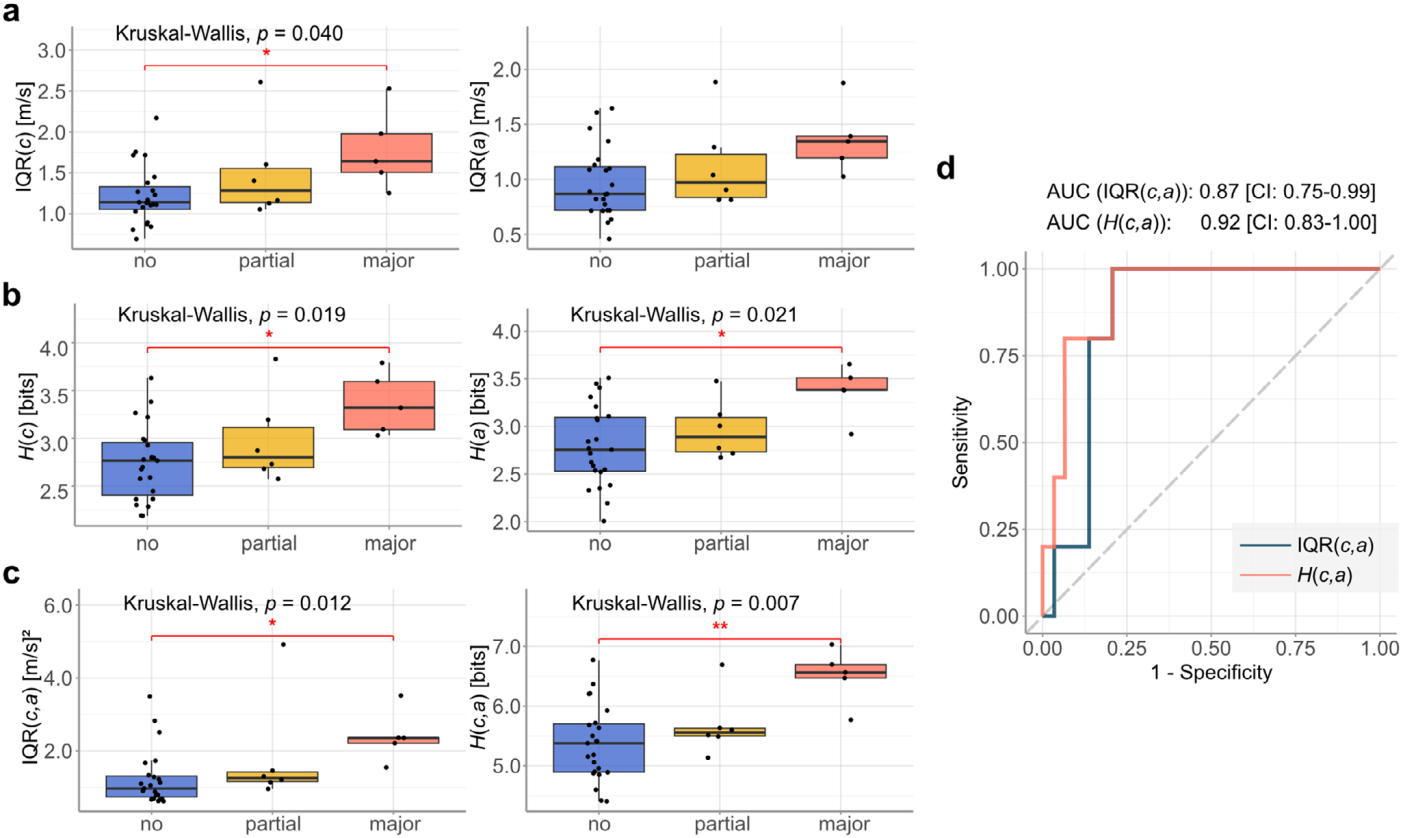
Intratumoral biomechanical heterogeneity in CRLM across treatment response groups. (a) IQR and (b) Shannon entropy of biomechanical parameter maps quantifying heterogeneity of the measured biomechanical parameters (no, partial, major refer to the respective treatment responses). (c) Joint IQR and Shannon entropy of shear wave speed *c* and penetration rate *a* maps indicating combined heterogeneity and overall biomechanical complexity. (d) AUC analysis based on joint metrics of *c* and *a* for distinguishing major from non‐major (no and partial responses) responders. Asterisks indicate significance levels: ^*^ *p* <0.05, ^**^ *p* <0.01.

Group‐averaged parameters showed a trend toward lower *c* and *a* values in non‐ and partial responders compared with major responders (*c*: 6.0 ± 1.7 m/s versus 5.8 ± 0.8 m/s versus 8.4 ± 2.1 m/s, *p* = 0.071; *a*: 3.0 ± 1.0 m/s versus 2.8 ± 0.4 m/s versus 4.5 ± 1.2 m/s, *p* = 0.069). Since specimens from partial responders still contained substantial amounts of viable tumor cells (as evidenced by histopathology), indicating incomplete chemotherapy, we pooled partial and non‐responders into a single group of *non‐major response* to clearly distinguish between patients with successful treatment and those without. Both group‐averaged parameters *c* and *a* were lower in non‐major responders compared with major responders (*c*: 6.0 ± 1.5 m/s versus 8.4 ± 2.1 m/s, *p* = 0.022; *a*: 3.0 ± 0.9 m/s versus 4.5 ± 1.2 m/s, *p* = 0.019). Biomechanical heterogeneity analysis revealed consistent differences across both single‐ and joint‐parameter metrics (Figure 6a–c). The IQR of *c* was lower in non‐ and partial responders than in major responders (Figure 6a). Similarly, the Shannon entropies of *c* and *a* showed an increased distributional randomness in major responders (Figure 6b). These trends were confirmed by the joint metrics, where both joint IQR and joint Shannon entropy increased progressively from non‐ and partial to major responders (Figure 6c). After pooling, joint IQR showed that CRLM samples were more biomechanically homogeneous in non‐major responders than major responders (1.4 ± 0.1 m^2^/s^2^ versus 2.4 ± 0.7 m^2^/s^2^, *p* = 0.007), similar to joint Shannon entropy (5.4 ± 0.6 bits versus 6.5 ± 0.5 bits, *p* = 0.001). For diagnosis, area under the receiver operating characteristic curve (AUC) analysis for differentiating major responders from non‐major responders gave an AUC of 0.82 [CI: 0.53–1.00] for *c* with a Youden index cutoff of 5.0 m/s versus an AUC of 0.83 [CI: 0.59–1.00] for *a* with a cutoff of 2.7 m/s. In terms of heterogeneity, AUC was 0.87 [CI: 0.75–0.99] for joint IQR with a cutoff of 1.5 m^2^/s^2^ and 0.92 [CI: 0.83–1.00] for joint Shannon entropy with a cutoff of 5.8 bits (Figure 6d). Complete results of group comparisons and AUC analysis are compiled in Table 1. For each CRLM sample, the histogram of pixel‐wise values from *c* and *a* maps and the 2D joint histogram of paired *c* and *a* values are presented in Figures S4, S5, and S6, respectively. The histological heterogeneity of CRLM samples was reflected by various features such as variable fractions of vital tumor cells, necrosis, fibrosis, and stromal components. These have been quantified by a pathologist and are reported for all samples in Note S2. Notably, samples from major responders exhibited higher necrotic fractions compared to non‐major responders (*p* = 0.0005, Figure S3). This observation is consistent with the increased biomechanical heterogeneity detected by ttMRE, suggesting that necrotic regions contribute substantially to the biomechanical variability observed within the lesions.

**TABLE 1:** Statistical results and AUC analysis of biomechanical parameter maps and corresponding biomechanical heterogeneity metrics of CRLM tumor samples across treatment response groups.

	Biomechanical Mapping Parameters		Biomechanical Heterogeneity Metrics					
			IQR		Joint IQR	Shannon Entropy		Joint Shannon Entropy
	*c* [m/s]	*a* [m/s]	*c* [m/s]	*a* [m/s]	IQR(*c*, *a*) [m/s]^2^	H(*c*) [bits]	H(*a*) [bits]	H(*c*, *a*) [bits]
Non‐responders	6.02 ± 1.65	3.02 ± 0.98	1.22 ± 0.35	0.96 ± 0.32	1.24 ± 0.76	2.74 ± 0.39	2.79 ± 0.42	5.37 ± 0.63
Partial responders	5.82 ± 0.75	2.80 ± 0.43	1.49 ± 0.58	1.12 ± 0.41	1.83 ± 1.52	2.98 ± 0.47	2.96 ± 0.31	5.68 ± 0.53
Major responders	8.36 ± 2.13	4.48 ± 1.24	1.78 ± 0.49	1.37 ± 0.32	2.40 ± 0.71	3.37 ± 0.32	3.37 ± 0.28	6.51 ± 0.46
Non‐major responders^a^	5.98 ± 1.50	2.98 ± 0.89	1.28 ± 0.41	1.00 ± 0.34	1.36 ± 0.96	2.79 ± 0.41	2.83 ± 0.40	5.43 ± 0.61
*p*‐value for a Kruskal–Wallis test among non‐, partial, and major responders	−	−	**0.040**	−	**0.012**	**0.019**	**0.021**	**0.007**
Post‐hoc analysis^b^	−	−	**0.041**	−	**0.014**	**0.016**	**0.018**	**0.005**
*p*‐value between non‐major and major responders	**0.022**	**0.019**	**0.019**	**0.029**	**0.007**	**0.006**	**0.007**	**0.001**
AUC between non‐major and major responders	0.82 [0.53–1.00]	0.83 [0.59–1.00]	0.83 [0.67–0.99]	0.81 [0.64–0.97]	0.87 [0.75–0.99]	0.88 [0.76–1.00]	0.87 [0.71–1.00]	0.92 [0.83–1.00]
Cutoff	5.00	2.70	1.24	0.99	1.50	3.01	2.89	5.75
Sensitivity	1.00 [1.00–1.00]	1.00 [1.00–1.00]	1.00 [1.00‐1.00]	1.00 [1.00–1.00]	1.00 [1.00–1.00]	1.00 [1.00–1.00]	1.00 [1.00–1.00]	1.00 [1.00–1.00]
Specificity	0.24 [0.10–0.41]	0.38 [0.21–0.55]	0.62 [0.45‐0.79]	0.59 [0.41–0.76]	0.79 [0.66–0.93]	0.79 [0.62–0.93]	0.62 [0.45–0.79]	0.79 [0.66–0.93]

^a^ Partial‐ and non‐responders were pooled into a single group of non‐major responders to clearly distinguish between patients with successful treatment and those without.

^b^ Post‐hoc *p*‐values are reported only for pairwise comparisons between non‐responders and major responders, as these were the only subgroups showing significant differences.

Statistically significant differences are indicated in bold.

Non‐significant differences are indicated by “‐”.

## 3 Discussion

In this study, we address a long‐standing challenge in elastography and biomechanics: capturing stiffness and viscosity maps within the bulk of heterogeneous soft tissue specimens traditionally requires high spatial resolution and a small voxel size. While small animal MRI scanners at 7 or 9.4 Tesla can provide high‐resolution MRE images, they depend on costly infrastructure and often require long acquisition times due to prolonged relaxation times at high fields [22, 32]. To overcome this challenge, we here propose to constrain multifrequency wave inversion of cylindrical shear waves in ttMRE with rheological priors derived from analytic Bessel solutions. Our method merges compact, low‐cost instrumentation with spatially resolved mapping, yielding bulk biomechanical parameters *c*, *a*, and *φ* at 150 µm pixel resolution for biomechanical heterogeneity analysis. As shown in CRLM specimens from cancer patients, marked heterogeneity allowed a clear separation of non‐major from major responders and therefore has the potential to serve as a cost‐effective biomarker of chemotherapy responsiveness.

Numerical wave simulations gave insight into the performance and limitations of the proposed spatially resolved inversion framework under controlled conditions. Our analysis indicated that the achievable resolution is governed by the interplay between excitation frequency, inclusion size, and mechanical contrast. Inclusions were better detectable with softer properties than the matrix and using higher frequencies, highlighting the importance of appropriate frequency selection for resolving submillimeter mechanical heterogeneity. In addition, our phantom experiments confirmed that ttMRE mapping reproduces reference values. In a homogeneous phantom with viscoelastic properties similar to human liver, all parameters matched values previously reported by Morr et al., [28]. In heterogeneous phantoms, ttMRE reliably detected inclusions and yielded consistent stiffness and viscosity estimates in materials mimicking soft biological tissue. Applied to heterogeneous CRLM samples, this method reproduced the key findings reported by Skrip et al. [20], namely that specimens from major responders exhibited higher stiffness and a more elastic‐solid behavior than those from non‐major responders. Importantly, ttMRE now enables pixel‐resolved mapping, allowing detailed scrutiny of these previously coarse‐grained rheological responses.

Tumor heterogeneity is a hallmark of cancer across genetic, biophysical, and clinical levels, including biomechanical signaling and structures [29, 33, 34]. Biomechanical heterogeneity arises from a variety of factors, such as cellular phenotype and extracellular matrix (ECM) deposition, which in turn leads to fibrosis, cellular clustering, cellular unjamming, necrosis, interstitial fluid accumulation, and aberrant vascularization [4, 6, 35, 36]. As these factors vary considerably between lesions that exhibit different responses to chemotherapy [37], biomechanical heterogeneity may help physicians to predict treatment outcome. In the CRLM samples investigated here, we found higher heterogeneity in major responders than in non‐major responders with excellent diagnostic accuracy. As confirmed by the presented histological heterogeneity analysis, chemotherapy‐induced tissue remodeling can introduce regional mechanical variability [38]. In major responders, treatment often leads to necrosis, fibrosis, and residual viable tumor islands [20, 39, 40, 41] collectively shaping a biomechanical environment characterized by higher stiffness, reduced viscosity, and prominent biomechanical heterogeneity. In particular, the increased necrotic fractions observed in major responders are consistent with the increased biomechanical heterogeneity detected by ttMRE. Remarkably, Chang et al., [42]. distinguished conventional necrosis in chemonaïve CRLM from infarct‐like necrosis associated with treatment response, demonstrating that the latter was associated with improved disease‐free survival and treatment. This supports the notion that chemotherapy‐induced necrosis is a key feature of treatment response and may contribute to the biomechanical heterogeneity observed in our data. In contrast, non‐responders often retain more uniform, viable tumorous tissue with less structural disruption [20], resulting in relatively homogeneous biomechanical profiles. While stiffness and viscosity features may overlap in responders and non‐responders, heterogeneity seems to be a unique feature of chemotherapy responsiveness.

Our study identifies biomechanical heterogeneity in CRLM as a promising in vivo biomarker for monitoring systemic therapy response using clinical MRE. Current advances in multiplex MRE techniques, which improve spatial resolution, strengthen this translational perspective [43]. At present, there is a critical need to reconcile discrepancies between radiological findings and pathological response [44], which are strongly linked to disease‐free and overall survival [45] but typically require invasive biopsy. Radiomic features such as entropy have been proposed as response markers [46], and MRE has already been used for treatment monitoring in CRLM patients [47]. Extending beyond this state of the art, our joint entropy parameters provide the first reference suggesting that biomechanical heterogeneity has the potential to become a discriminative marker in clinical MRE and, after surgery, in spatially resolved ttMRE.

Despite these advances, our study has limitations. First, validation experiments significantly varied in agar‐based heterogeneous phantoms. This is likely attributable to the almost purely elastic behavior of agar [48], in which shear waves can propagate nearly unattenuated. This low attenuation behavior is clearly beyond our detection limits for viscosity within the explored spatial‐dynamic regime of ttMRE. Similarly, related to damping was the varying level of wave amplitudes between heterogeneous and homogeneous phantoms that may have affected the accuracy in some of our ground‐truth scenarios [49]. Moreover, gelatin‐based phantoms may have been subject to variability introduced during preparation, as pouring warm gelatin solution into the pre‐solidified gelatin matrix can partially remelt the matrix boundaries. Second, the CRLM sample size was small, particularly for the major and partial response groups, which may have impacted the generalizability of the statistical results. Therefore, our ROC analysis should be interpreted as exploratory, and the results require validation in larger cohorts. Third, biomechanical parameters were derived from a single axial slice positioned in the central region of the sample. As this approach is blind to heterogeneity along the longitudinal direction, the heterogeneity of tumor samples may have been underestimated. Future studies incorporating three‐dimensional biomechanical mapping may enable a more comprehensive assessment of tumor heterogeneity and improve characterization of spatially varying tissue properties. Fourth, we addressed ex vivo biomechanical parameters, which naturally differ from in vivo properties due to the absence of blood perfusion and reduced solid stress [6, 50]. Furthermore, ttMRE uses a higher frequency range than clinical MRE, making it difficult to directly compare in vivo and ex vivo properties. Therefore, viscoelastic model parameters such as provided by the spring‐pot model are needed for frequency‐independent assessment and comparability with clinical data. Finally, to substantiate biomechanical–histological relationships, future studies should perform co‐registration of biomechanical parameter maps with H&E‐stained sections, allowing direct correlation between local viscoelastic parameters and tissue morphology.

## 4 Conclusion

In conclusion, we demonstrate the feasibility of mapping biomechanical properties in ex vivo tumor samples using ttMRE. Biomechanical heterogeneity—quantified by the joint Shannon entropy of shear wave speed and penetration rate—correlates with the response to chemotherapy in patients with CRLM, providing a diagnostic marker of cancer cell viability. Image‐resolved ttMRE may become a cost‐effective tool for biomechanical profiling of resected tumor specimens, enabling automated assessment of cell viability and therapy responsiveness.

## 5 Methods

### 5.1 Wave Simulations

Wave simulations were conducted to estimate the effective spatial resolution limits of spatially resolved ttMRE. We used the staggered‐grid compact finite‐difference stencil for wave modeling, which was originally proposed by Jo et al., [51]. building on Virieux's staggered‐grid formulation [52]. More recently, Aghamiry et al., [53]. made the stencil weights adaptive to the local wave speed for each voxel by solving a dispersion minimization problem prior to wave simulation. This method has been validated for seismological applications [54] and medical imaging on ex vivo and in vivo experiments [55].

To approximate the wave fields generated in ttMRE, 360 azimuthally equidistant point sources were distributed along a circular boundary with a radius of 3.5 mm. When the sources were not located exactly on the grid points, Kaiser‐windowed sinc functions were used to distribute them onto the neighboring grid points [56]. The simulation grid size was 64 × 64 with a spatial resolution of 0.15 × 0.15 mm^2^. For cases where wavelengths were supported by fewer than seven pixels, the grid was refined to 128 × 128 with a resolution of 0.075 × 0.075 mm^2^ to ensure numerical accuracy. Complex‐valued Gaussian noise was added to simulate a displacement signal‐to‐noise ratio (SNR) of approximately 28 dB according to Donoho et al., [57]. The background matrix was defined using the spring‐pot model with a shear modulus *µ* = 33 kPa and a power law exponent *α* = 0.24, while the viscosity *η* was fixed at 1 Pa∙s. These parameters were chosen to mimic a 25 wt.% gelatin gel material in the gelatin‐based phantom.

Two circular inclusions were embedded symmetrically along the horizontal axis, with a center‐to‐center distance of 1.5 mm around the sample center. The inclusions exhibited opposite shear wave speed *c* contrasts: the left inclusion had a negative contrast, while the right inclusion had a positive contrast. The *c* contrast was defined as:

(1) $$contrast=\frac{mean\left(inlusion\right)-mean\left(background\;matrix\right)}{mean\left(background\;matrix\right)}\;,$$

where *mean(inclusion)* and *mean(background matrix)* were calculated as the average *c* values within ROIs corresponding to the inclusion and surrounding background matrix, respectively. The inclusions were modeled as circular regions with Gaussian‐distributed complex‐valued wavenumber profiles. Their sizes ranged from 0.45 to 2.55 mm in increments of 0.15 mm. The absolute *c* contrast varied from 15% to 80% in steps of 5% relative to the background, with 15% defined as the lower bound for detectable contrast.

To evaluate frequency‐dependent resolution effects, simulations were conducted over a frequency range of 1100 Hz to 5000 Hz in steps of 300 Hz. The lower bound of 1100 Hz was selected such that the longest wavelength did not exceed the sample diameter [24]. For each condition, complex‐valued wave fields from three consecutive frequencies were used as input to the multifrequency spatially resolved postprocessing pipeline. The *c* contrast of the inclusion relative to the background *c* was determined and compared with the underlying true contrast of the simulation to generate contour error maps depending on inclusion size, true contrast, and center frequency of inversion. Notably, the evaluation of effective spatial resolution was performed exclusively on the reconstructed *c* maps, as *c* provides the most direct and robust metric for assessing inclusion detectability in this framework [25, 58]. Finally, the effective spatial resolution limits were derived from these contour maps by defining inclusions as detectable when the reconstructed absolute contrast exceeded 20%.

### 5.2 Phantoms and Tissue Specimens

Three phantoms with different viscoelastic properties and degrees of heterogeneity were investigated: 1) a homogeneous PAAm mimicking the viscoelastic properties of healthy in vivo liver [28], 2) a mainly elastic agar‐based phantom with three cylindrical inclusions, and 3) a viscoelastic gelatin‐based phantom with two cylindrical inclusions.

The viscoelastic phantom 1) was made of non‐crosslinked PAAm, as described in detail by Morr et al., [28]. The elastic phantom 2) was made of 1.8 wt.% agar (Merck KGaA, Darmstadt, Germany) as matrix material with three 3‐mm inclusions. The inclusions were composed of ultrasound gel (Medimex GmbH, Limburg, Germany), 0.5 wt.% agar, and 1.2 wt.% agar, respectively. The viscoelastic phantom 3) was made of 25 wt.% gelatin (Sigma‐Aldrich, St. Louis, USA) as matrix material containing two 2‐mm inclusions. The inclusions were made of 10 and 35 wt.% gelatin. Additionally, a series of homogeneous phantoms with agar, gelatin, and ultrasound gel at the aforementioned concentrations were prepared as reference standards. Detailed protocols for the preparation of agar‐ and gelatin‐based phantoms are provided in Notes S3 and S4. The mechanical properties of all materials were independently characterized using shear oscillatory rheometry, as described in Note S1.

Thirty‐four CRLM samples from 31 patients who underwent liver resection and gave informed consent were investigated. The tissue samples were collected between May 2022 and May 2023 in the Department of Surgery of Charité—Universitätsmedizin Berlin (Campus Charité Mitte and Campus Virchow Klinikum) under institutional approval (EA1/214/19 and EA4/132/22). The original lesion sizes were not systematically recorded; however, due to the requirements of the ttMRE setup, all analyzed samples had minimum dimensions of approximately 8 × 8 × 3 mm^3^ (width × depth × height). All samples were histologically graded for cancer regression grading according to Rubbia–Brandt [59]. In addition, histological composition reflecting intratumoral heterogeneity, including fractions of vital tumor cells, necrosis, fibrosis, and stromal components, was quantitatively assessed by a board‐certified pathologist. This information is provided in Note S2 for all samples. Details of the patient population, tissue handling, and histological assessment have been reported previously [20].

### 5.3 ttMRE Setup and Acquisition

ttMRE was performed in a benchtop MRI scanner with a 0.5 Tesla permanent magnet (Magspec, Pure Devices GmbH, Würzburg, Germany), a four‐channel external gradient amplifier (DC600, Pure Devices GmbH, Würzburg, Germany), and a piezoelectric actuator (PAHL60/20, Piezosystem Jena GmbH, Jena, Germany). The actuator generated mechanical vibrations primarily polarized along the tube's longitudinal *z*‐axis (principal cylinder axis). A ttMRE setup is provided in Figure 1. Samples were placed and imaged in cylindrical glass tubes (inner diameter: 7 mm, outer diameter: 9 mm, length: 200 mm). To ensure sufficient wave amplitude along the principal cylinder axis, a PVC foam disk was placed between the sample and the plug that sealed the bottom of the tube. Shear wave images were acquired using a spin‐echo sequence with trapezoidal bipolar motion‐encoding gradients (MEGs) applied along the z‐axis, with amplitudes ranging from 0.2 to 0.4 T/m and a variable number of MEG cycles depending on the vibration frequency and MEG duration [24].

One axial slice of 3 mm thickness and 0.15 × 0.15 mm^2^ pixel resolution (matrix size of 64 × 64) was acquired at four equidistant time steps over a wave cycle. The imaging slice was positioned in the near central region of the cylindrical sample to minimize boundary effects from the top and bottom surfaces and to ensure a more spatially uniform wave field. Mechanical vibrations in a range of 500 to 5300 Hz were consecutively applied and acquired. For phantom samples, vibration frequencies were selected based on the mechanical properties of the materials to ensure stable wave propagation with sufficient signal amplitude and adequately resolved wavelengths. Frequencies with insufficient wave penetration were excluded based on a wave attenuation criterion derived from the imaginary part of the wavenumber (see Note S5). For phantom samples, vibration frequencies were optimized to achieve the best wave field SNR. Based on each sample's T1/T2 relaxation times, echo time (TE), repetition time (TR), and duration and amplitude of the MEGs were individually optimized. Detailed acquisition parameters are provided in Table S5.

### 5.4 ttMRE Postprocessing

The acquired ttMRE phase data were processed using a previously established Bessel‐fit‐based algorithm to inform spatially‐resolved multifrequency wave‐inversion mapping [25], as illustrated in Figure 2. The Bessel‐fit pipeline assumes azimuthal symmetry and homogeneity of the sample, yielding global, frequency‐resolved scalar estimates of shear wave speed *c* and shear wave penetration rate *a* for deriving sample‐averaged viscoelastic parameters as priors for an automated bandpass setup employed in the subsequent wave inversion. Multifrequency wave inversion combined frequency‐adaptive, spatial‐directional filters with phase‐gradient wavenumber recovery as detailed below.

### 5.5 Bessel‐Fit‐Based Postprocessing

After phase unwrapping [60] and temporal Fourier transform, the shear wave field was fit with an analytical Bessel function solution [24] to obtain complex‐valued wavenumber k^*^:

(2) $$k^{*}=k^{\prime }+ik^{\prime \prime }.$$

Shear wave speed *c* and penetration rate *a* were derived by

(3) $$c=\frac{\omega }{k^{\prime }}\;,a=-\frac{\omega }{2\pi k^{\prime \prime }}\;.$$

Frequency‐dependent values of *c* and *a* were further fit with a spring‐pot rheological model [26] to derive frequency‐independent viscoelastic parameters. The workflow is illustrated in Figure 2a.

### 5.6 Spatially Resolved Postprocessing

To reduce noise while preserving structural details, the raw complex‐valued ttMRE signal was smoothed in the wavenumber domain with a Gaussian low‐pass filter of 0.05 mm standard deviation, followed by masking, unwrapping, and temporal Fourier transform to obtain the complex‐valued wave field at each frequency *f*. Bandpass directional filters, consisting of a Butterworth spatial radial bandpass and a cos^2^‐shaped directional component [61], were then applied in the wavenumber domain to isolate unidirectional plane shear wave components propagating along eight directions [25].

Butterworth bandpass filters consisting of a third‐order low‐pass filter with a threshold of 1.6 × 10^4^ m^−1^ for effective smoothing while preserving low‐wavelength components, and a first‐order high‐pass component. The high‐pass thresholds were selected according to the outcome of the Bessel fits. A Bessel‐fit error (BFE) empirical threshold of 0.8 rad was used (see Note S6). For samples with a BFE greater than 0.8, the threshold was set based on the wavelength assessed directly from the wave images. Conversely, for samples with BFEs lower than 0.8 – whose wave patterns were predominantly concentric due to the cylindrical glass tube and could therefore be considered quasi‐homogeneous – an iterative approach was implemented to enable automated selection of high‐pass thresholds. All candidate high‐pass values were iteratively tested and optimized by minimizing the difference between the reconstructed values and Bessel‐fit references.

For each propagation direction ${\left\{d_{j}\right\}}_{j=1}^{8}$ and frequency ${\left\{f_{k}\right\}}_{k=1}^{m}$, the plane shear wave field was modeled as:

(4) $$u_{d_{j}f_{k}}^{*}\;\left(r,t\right)=A\cdot e^{i\left(k^{*}r-\omega_{k}t\right)},$$

where ω_*k*_ =  2π*f_k_* is the angular frequency. The real and imaginary parts of the wavenumber were deduced from the phase gradient [61] and the amplitude gradient [25] of $u_{d_{j}f_{k}}^{*}$, respectively:

(5) $${k^{\prime }}_{d_{j}f_{k}}=\left\|\nabla arg\left(u_{d_{j}f_{k}}^{*}\left(r,t\right)\right)\;\right\|,$$

(6) $${k^{\prime \prime }}_{d_{j}f_{k}}=\left\|\frac{\nabla \left|u_{d_{j}f_{k}}^{*}\left(r,t\right)\right|}{\left|u_{d_{j}f_{k}}^{*}\left(r,t\right)\right|}\right\|\;.$$

Finite gradients were computed using the Anderssen gradient scheme with 2D‐multidimensional kernels (a kernel size of 3 pixels for phase, and 5 pixels for amplitude to account for the higher noise impact on amplitude) and weighted averaging to further suppress noise [62]. To avoid overestimation caused by short waves with few supporting pixels, as analyzed in Mura et al. [63], Lanczos3 interpolation with a factor of 2 was applied to the raw ttMRE data when the local wavelength, estimated from radial wave profiles across the wave field, was supported by less than 7 pixels.

Finally, frequency‐averaged parameter maps were computed as wave amplitude‐weighted averaging over directions, followed by weighted averaging across all frequencies:

(7) $$c=\frac{\sum_{k=1}^{m}\left(\frac{\omega_{k}\sum_{j=1}^{8}w_{d_{j}f_{k}}}{\sum_{j=1}^{8}k_{d_{j}f_{k}}^{\prime }w_{d_{j}f_{k}}}w_{f_{k}}\right)}{\sum_{k=1}^{m}w_{f_{k}}},$$

(8)

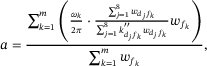

with $w_{d_{j}f_{k}}=\left|u_{d_{j}f_{k}}^{*}\left(r,t\right)\right|$ as the empirical directional weighting term and $w_{f_{k}}=\sum_{j=1}^{8}w_{d_{j}f_{k}}$ denoting the total directional weighting across directions at each frequency.

To obtain a measure of relative viscosity (referred to in the literature as tissue fluidity or friction [50]), the loss angle, *φ*, was derived as: [24]

(9) $$\varphi =2\cdot tan^{-1}\left(\frac{c}{2\pi \cdot a}\;\right)\;.$$

All spatially resolved postprocessing parameters for the phantoms are listed in Table S2. Since BFEs were below 0.8 rad for all ex vivo CRLM tissue samples, high‐pass thresholds were iteratively optimized, ranging from 63 to 2627 m^−1^ for all tissue samples. To avoid boundary artifacts introduced by spatial filtering [64], evaluation was restricted to the central 5.6 mm region of the 7 mm diameter sample. Frequencies for mapping biomechanical parameters of CRLM tissue samples were selected to ensure sufficient wave propagation throughout the imaging plane for specimens covering a broad stiffness range, with optimal quality achieved at 1700, 2100, and 2500 Hz.

### 5.7 Quantification of Biomechanical Heterogeneity

Biomechanical heterogeneity of the samples was assessed using two complementary metrics: IQR and Shannon entropy [29, 30, 31]. IQR served as a nonparametric measure of spread, capturing the variability in each parameter map across all pixels:

(10) $$IQR\left(X\right)=Q_{3}\;\left(X\right)-Q_{1}\left(X\right),$$

where *Q_3_* and *Q_1_* are the 75th and 25th percentiles of the pixel values in the parameter map X∈{c,a,φ}, respectively. In addition, the product of IQR(*c*) and IQR(*a*) was calculated to describe the combined spread of stiffness and viscosity, denoted as the joint IQR(*c*, *a*).

Shannon entropy was used to quantify distributional heterogeneity (i.e., uncertainty or randomness) across parameter maps:

(11) $$H\;\left(X\right)=-\sum_{i=1}^{N}p_{i}\log_{2}p_{i},$$

where *p_i_* is the probability of observing the pixel value falling into the *i*‐th bin of a histogram constructed from each parameter map *X*, and *N* is the total number of bins [31].

To capture the multidimensional distributional heterogeneity between two parameters, we calculated the joint Shannon entropy of *c* and *a* as:

(12) $$H\;\left(c,a\right)=-\sum_{i=1}^{N}\sum_{j=1}^{N}p_{ij}\log_{2}p_{ij},$$

where *p_ij_* is the joint probability of observing values from *c* and *a* maps falling into the *i*‐th and *j*‐th bins of their respective histograms [31]. For all entropy calculations, histogram ranges were chosen to encompass the approximate value ranges observed in the parameter maps (0‐16 m/s for *c* and 0–12 m/s for *a*), with the number of bins empirically set to *N* = 30. The fixed ranges and number of bins avoid introducing biases that can arise from data‐dependent binning methods and ensure sufficient resolution for estimating probabilities while maintaining statistical stability across parameter maps, thereby allowing consistent entropy calculations across all maps and subjects.

### 5.8 Statistics

Statistical analysis was performed in R (version 4.4.3) and RStudio (2025.05.1, Posit PBC, Boston, US). Group comparisons of mean values and biomechanical heterogeneity metrics across the three histological response groups were conducted using the Kruskal–Wallis test, followed by Dunn's tests with Bonferroni correction for pairwise post‐hoc analysis. Group values were reported as mean and standard deviation. Additionally, Wilcoxon rank‐sum tests were used for binary comparison of non‐major (partial and non‐responders combined) versus major responders (successful treatment). Receiver operating curve (ROC) analysis was performed to assess the discriminatory power for distinguishing major from non‐major responders. AUC, sensitivity, and specificity with 95% confidence interval (CI), and optimized cutoff values using the Youden index were computed to quantify classification performance. A *p*‐value <0.05 was considered significant.

## Funding

This study was supported by the German Research Foundation (DFG, FOR5628, CRC1340, GRK2260 BIOQIC) and the Berliner Krebsgesellschaft (PAFF202131). J. Pratschke, I. M. Sauer, I. Sack were financially supported by Cluster of Excellence Matters of Activity, Image Space Material funded by the Deutsche Forschungsgemeinschaft (DFG, German Research Foundation) under Germany's Excellence Strategy—EXC 2025 – 390648296, Berlin, Germany.

## Conflicts of Interest

The authors declare no conflicts of interest.

## Supporting information




**Supporting File**: advs75728‐sup‐0001‐SuppMat.docx.

## Data Availability

The data that support the findings of this study are available from the corresponding author upon reasonable request.
