# Supplementary material for: Compact Tabletop Magnetic Resonance Elastography for Mapping Soft Tissue Viscoelasticity
Source: Adv Sci (Weinh). 2026 May 19;13(45):e75728. doi: 10.1002/advs.75728 (PMC13335987; doi:10.1002/advs.75728)
Supplement: Supplementary file 1 [file ADVS-13-e75728-s001.docx]

Supporting Information

Compact tabletop magnetic resonance elastography for mapping soft tissue viscoelasticity

*Weijie Zhao, Lisa-Marie Skrip, Heiko Tzschätzsch, Tom Meyer, Hossein S. Aghamiry, Alexander Arnold, Lene Änne Böhne, Jakob Jordan, Oliver Boehm, Agnes K. Boehm, Steffen Görner, Jakob Schattenfroh, Yanglei Wu, Pedro Augusto Dantas de Moraes, Johann Pratschke, Jürgen Braun, Igor M. Sauer, Jing Guo, Ingolf Sack*, Karl H. Hillebrandt*

**Table of Content**

[Supplementary Note 1: Rheometry validation for phantom materials	1](#_Toc227188365)

[Supplementary Table 1: Postprocessing parameters, biomechanical properties, and heterogeneity metrics for phantom experiments	6](#_Toc227188366)

[Supplementary Note 2: Histological heterogeneity of all CRLM samples	7](#_Toc227188367)

[Supplementary Figure 1: Histograms of pixel-wise shear wave speed *c* values per CRLM sample across treatment response groups	9](#_Toc227188368)

[Supplementary Figure 2: Histograms of pixel-wise penetration rate *a* values per CRLM sample across treatment response groups	10](#_Toc227188369)

[Supplementary Figure 3: Joint histograms of pixel-wise shear wave speed *c* and penetration rate *a* values per CRLM sample across treatment response groups	11](#_Toc227188370)

[Supplementary Note 3: Heterogeneous elastic agar-based phantom preparation	11](#_Toc227188371)

[Supplementary Note 4: Heterogeneous viscoelastic gelatin-based phantom preparation	12](#_Toc227188372)

[Supplementary Note 5: Frequency selection and wave attenuation-based exclusion criterion	13](#_Toc227188373)

[Supplementary Table 2: ttMRE acquisition parameters for phantom experiments	13](#_Toc227188374)

[Supplementary Note 6: Bessel-fit error	14](#_Toc227188375)

[Supplementary References	15](#_Toc227188376)

## **Supplementary Note 1: Rheometry validation for phantom materials**

To evaluate whether ttMRE can serve as a reliable tool for quantifying viscoelastic properties in small soft tissue samples, all phantom materials were additionally characterized using shear oscillatory rheometry. Measurements were performed using a rheometer (MCR 301, Anton Paar, Graz, Austria) equipped with a parallel plate measuring system (PP50, Anton Paar, Graz, Austria) over a frequency range of 10–60 Hz with the step of 5 Hz at a controlled temperature of 24 °C.

Rheometry provides the storage modulus $G^{'}$ (Pa) and loss modulus $G^{''}$ (Pa) at each driving frequency $f$. These quantities were converted into shear wave-related parameters (shear wave speed *c*, penetration rate *a*) to enable comparison with ttMRE measurements:

$$c= \sqrt{\frac{2({G^{'}}^{2}+{G^{''}}^{2})}{\rho(\sqrt{{G^{'}}^{2}+{G^{''}}^{2}}+G^{'})}} ,$$

(S1)

$$a=\frac{1}{2\pi}\sqrt{\frac{2({G^{'}}^{2}+{G^{''}}^{2})}{\rho(\sqrt{{G^{'}}^{2}+{G^{''}}^{2}}-G^{'})} ,}$$

(S2)

where $\rho$ denotes the material density, assumed to be 1000 kg/m^3^.

For gelatin phantom materials, a fractional Kelvin-Voigt with one parallel spring-pot model^[1]^ was used:

$$G^{*}= {\mu_{1}}^{1-\alpha}{(i\omega\eta)}^{\alpha}+ \mu_{2} ,$$

(S3)

where $G^{*}$ is the complex shear modulus, $\mu_{1}$, $\eta$ and $\alpha$ represent the spring-pot parameters (shear modulus, viscosity, and power law exponent), and $\mu_{2}$ is the shear modulus of the parallel spring component. The angular frequency is given by $\omega=2\pi f$. The viscosity parameter $\eta$ was fixed at 1 Pa∙s.

For all other phantom materials, a spring-pot model was applied:

$$G^{*}= \mu^{1-\alpha}{(i\omega\eta)}^{\alpha},$$

(S4)

with fixed $\eta$.

Model fitting was performed using rheometry data acquired at lower frequencies. The resulting frequency-independent parameters were then used to extrapolate $G^{*}$ to the higher frequency range corresponding to ttMRE measurements.

**Figure S1** and **S2** present the rheometry data alongside the fitted model curves and corresponding ttMRE measurements. All fitted parameters for each material are summarized in **Table S1**.

**Figure S1.** Comparison of rheometry-derived and ttMRE-measured wave parameters across phantom materials. Shear wave speed (c, blue) and penetration rate (a, red) are shown for linear polymerized polyacrylamide (PAAm), ultrasound (US) gel, and agar gels (0.5, 1.2, and 1.8 wt%). Solid lines with open markers represent values converted from rheometry at lower frequencies, while dashed/dotted lines indicate model fits based on Equation S4 extrapolated to higher frequencies. Filled markers denote ttMRE measurements acquired at higher frequencies. Different marker shapes correspond to different materials.

**Figure S2.** Rheometry-based model fitting and ttMRE comparison for gelatin phantom materials. Shear wave speed (c, blue) and penetration rate (a, red) are shown for gelatin gels (10, 25, and 35 wt%). Solid lines with open markers represent rheometry-derived values at lower frequencies, while dashed/dotted curves were fitted based on Equation S3 (fractional Kelvin–Voigt model) and extrapolated to higher frequencies. Filled markers indicate ttMRE measurements. Marker shapes distinguish different gelatin concentrations.

**Table S1.** Rheological model parameters and quantitative agreement between rheometry-based predictions and ttMRE measurements for all phantom materials. Parameters were obtained by fitting either a spring-pot model or a fractional Kelvin–Voigt model (Equation 3 or 4) to rheometry data. The fitted models were used to extrapolate shear wave speed (c) and penetration rate (a) to the higher frequency range of ttMRE. Agreement between model predictions and ttMRE measurements was quantified using the root-mean-square error (RMSE) and percentage differences for c and a, respectively. The viscosity parameter $\eta$ was fixed where indicated.

| **Phantom** | **Material** | **Rheological model** | **Rheological parameters** | | | | **RMSE between fitted model and measured ttMRE c and a** | | **Percentage difference between fitted model and measured ttMRE c and a ^b)^** | |
| --- | --- | --- | --- | --- | --- | --- | --- | --- | --- | --- |
|  |  |  | $\boldsymbol{\mu/}\boldsymbol{\mu}_{\boldsymbol{1}}$ **[Pa]** | $\boldsymbol{\eta}$ **[Pa∙s]** | $\boldsymbol{\alpha}$ | $\boldsymbol{\mu}_{\boldsymbol{2}}$ **[Pa]** | **RMSE of c [m/s]** | **RMSE of a [m/s]** | $\boldsymbol{\delta}_{\boldsymbol{c}}$ **[%]** | $\boldsymbol{\delta}_{\boldsymbol{a}}$ **[%]** |
| Homogeneous viscoelastic PAAm | PAAm | Spring-pot | 2971 ± 14 | 7.3^a)^ | 0.438 ± 0.003 | / | 0.483 | 0.172 | -13.0 | 10.4 |
| Elastic agar-based | Ultrasound gel |  | 471 ± 14 | 1 | 0.178 ± 0.020 |  | 0.005 | 0.097 | 0.2 | -8.0 |
|  | 0.5 wt% agar gel |  | 1709 ± 56 |  | 0.048 ± 0.003 |  | 0.175 | 3.334 | 12.9 | 39.4 |
|  | 1.2 wt% agar gel |  | 8921 ± 102 |  | 0.070 ± 0.001 |  | 0.532 | 9.572 | 17.6 | 108.7 |
|  | 1.8 wt% agar gel |  | 4024 ± 937 |  | 0.123 ± 0.002 |  | 0.419 | 65.120 | 7.0 | 623.7 |
| Viscoelastic gelatin-based | 10 wt% gelatin gel | Fractional Kelvin-Voigt | 288 ± 38 |  | 0.607 ± 0.065 | 2312 ± 33 | 0.246 | 1.564 | 10.4 | 116.3 |
|  | 25 wt% gelatin gel |  | 28192 ± 6488 |  | 0.432 ± 0.026 | 14250 ± 385 | 0.666 | 0.289 | -10.8 | 2.7 |
|  | 35 wt% gelatin gel |  | 11996 ± 413 |  | 0.345 ± 0.011 | 22840 ± 174 | 0.501 | 3.018 | 8.1 | -33.1 |
| a) According to Morr *et al*.*.*^[2]^ In addition, the material density *ρ* was set to 1094 kg/m^3^ accordingly.  b) A positive percentage difference indicates overestimation, whereas a negative value indicates underestimation. | | | | | | | | |  |  |

In **Table S1**, assuming that the predicted rheological models adequately describe the materials behavior, for most materials, the root-mean-square error (RMSE) of the shear wave speed c remained below 0.7 m/s, and the percentage difference between fitted model predictions and measured ttMRE c was smaller than 18%, indicating consistent estimation of bulk stiffness. The penetration rate a also showed reasonable agreement in predominantly viscoelastic materials, such as PAAm and ultrasound gel, with percentage differences below 13%. In contrast, larger deviations in a were pronounced for agar phantom materials, with a maximum difference of 623.7%. Relatively small deviations in both c and a were observed for gelatin materials; however, for the 10 wt% gelatin gel, a exhibited a higher percentage difference of 116%.

A systematic deviation was further evident for agar phantom materials in **Figure S1**, where ttMRE tended to yield slightly higher c and substantially higher a values compared to rheometry-based predictions. This discrepancy could be partially attributed to limitations in rheometry measurements of agar gels. In particular, interfacial slippage between the sample and the rheometer plates might lead to an underestimation of the true mechanical stiffness, resulting in lower apparent moduli and consequently lower predicted wave parameters.^[3]^ In addition, MRE-derived parameters depend on displacement amplitude for agar gels,^[4]^ variations in wave amplitude may therefore contribute to an overestimation of mechanical parameters in ttMRE. For gelatin phantom materials (**Figure S2**), the observed deviations were relatively small and might partly arise from differences in measurement timing between the two techniques (rheometry performed within approximately 15 minutes after preparation, whereas ttMRE measurements were completed within about 1 hour). According to Fonkwe et al.,^[5]^ the viscoelastic properties of gelatin evolve over time during gelation, with changes in both storage and loss moduli, which may contribute to the observed differences.

Overall, despite differences in measurement principles and experimental conditions, both approaches consistently capture the relative variations in mechanical properties across materials. This demonstrates that the selected rheological models provide a meaningful bridge between low-frequency rheometry and high-frequency ttMRE measurements, and supports the use of ttMRE as a quantitative tool for characterizing viscoelastic properties.

## **Supplementary Table 1: Postprocessing parameters, biomechanical properties, and heterogeneity metrics for phantom experiments**

**Table S2.** Spatially resolved postprocessing parameters, biomechanical parameters derived from spatially resolved and Bessel-fit-based postprocessing pipelines, and biomechanical heterogeneity metrics obtained from mapping results across different phantoms.

| **Phantom** | | | Homogeneous viscoelastic PAAm | Heterogeneous elastic agar-based | | | | Heterogeneous viscoelastic gelatin-based | | |
| --- | --- | --- | --- | --- | --- | --- | --- | --- | --- | --- |
|  |  |  |  | I1: ultrasound gel | I2: 0.5 wt% agar gel | I3: 1.2 wt% agar gel | M1: 1.8 wt% agar gel | I4: 10 wt% gelatin gel | I5: 35 wt% gelatin gel | M2: 25 wt% gelatin gel |
| **Bandpass selection** | **BFE**  **[rad]** | | 0.16 ± 0.05 | 1.08 ± 0.16 | | | | 0.58 ± 0.10 | | |
|  | **Bandpass type** | | Butterworth | Butterworth | | | | Butterworth | | |
|  | **High-pass threshold**  **[m^-1^]** | | 487 – 2534 | 3000 | | | | 63 – 1270 | | |
|  | **Low-pass threshold**  **[m^-1^]** | | 16000 | 16000 | | | | 16000 | | |
| **Biomechanical parameters** | **Mapping** | ***c***  **[m/s]** | 2.95 ± 0.35 | 1.26 ± 0.06 | 1.48 ± 0.06 | 3.76 ± 0.06 | 5.25 ± 0.25 | 3.41 ± 0.24 | 7.22 ± 0.44 | 6.12 ± 0.30 |
|  |  | ***a***  **[m/s]** | 1.63 ± 0.14 | 1.25 ± 0.31 | 1.57 ± 0.31 | 7.07 ± 1.48 | 4.80 ± 0.60 | 2.37 ± 0.37 | 6.29 ± 1.63 | 5.60 ± 1.32 |
|  |  | ***φ***  **[rad]** | 0.56 ± 0.07 | 0.33 ± 0.07 | 0.31 ± 0.06 | 0.18 ± 0.04 | 0.35 ± 0.05 | 0.46 ± 0.08 | 0.38 ± 0.08 | 0.36 ± 0.09 |
|  | **Bessel-fit (from homogeneous reference sample)** | ***c***  **[m/s]** | 3.06 ± 0.30 | 0.94 ± 0.00 | 1.45 ± 0.00 | 3.56 ± 0.00 | 6.07 ± 0.00 | 2.58 ± 0.01 | 6.51 ± 0.03 | 5.53 ± 0.03 |
|  |  | ***a***  **[m/s]** | 1.73 ± 0.22 | 0.97 ± 0.00 | 8.07^a)^ ± 0.01 | 18.32^a)^ ± 0.01 | 70.47^a)^ ± 0.02 | 2.92 ± 0.01 | 6.05 ± 0.03 | 4.66 ± 0.02 |
|  |  | ***φ***  **[rad]** | 0.55 ± 0.02 | 0.30 ± 0.00 | 0.06 ± 0.00 | 0.06 ± 0.00 | 0.03 ± 0.00 | 0.28 ± 0.00 | 0.34 ± 0.00 | 0.37 ± 0.00 |
|  | **Percentage**  **difference** | ***c***  **[%]** | 3.66 ± 15.39 | 29.09 ± 4.66 | 2.05 ± 4.05 | 5.46 ± 1.59 | 14.49 ± 4.74 | 27.71 ± 6.91 | 10.34 ± 6.10 | 10.13 ± 4.92 |
|  |  | ***a***  **[%]** | 5.95 ± 15.33 | 25.23 ± 24.41 | 134.85 ± 10.77 | 88.62 ± 16.82 | 174.49 ± 2.99 | 20.79 ± 15.45 | 3.89 ± 25.91 | 18.32 ± 23.38 |
|  |  | ***φ***  **[%]** | 1.80 ± 13.02 | 9.52 ± 21.16 | 135.14 ± 10.52 | 100.00 ± 16.67 | 168.42 ± 4.16 | 48.65 ± 16.36 | 11.11 ± 20.99 | 2.74 ± 25.00 |
| **Biomechanical heterogeneity**  **metrics** | **IQR** | ***c***  **[m/s]** | 0.71 ± 0.02 | 2.25 ± 0.05 | | | | 1.18 ± 0.04 | | |
|  |  | ***a***  **[m/s]** | 0.22 ± 0.01 | 1.74 ± 0.07 | | | | 2.49 ± 0.08 | | |
|  | **Joint IQR** | **IQR(*c*, *a*)**  **[m/s]^2^** | 0.15 ± 0.01 | 3.92 ± 0.18 | | | | 2.93 ± 0.15 | | |
|  | **Shannon entropy** | **H(*c*)**  **[bits]** | 2.00 ± 0.02 | 3.06 ± 0.01 | | | | 2.79 ± 0.03 | | |
|  |  | **H(*a*)**  **[bits]** | 1.19 ± 0.02 | 3.65 ± 0.03 | | | | 4.05 ± 0.02 | | |
|  | **Joint Shannon entropy** | **H(*c*, *a*)**  **[bits]** | 3.10 ± 0.02 | 5.94 ± 0.01 | | | | 6.32 ± 0.03 | | |
| ^a)^ Due to the nearly non-attenuating property of agar gel. | | | | | | | | | | |
| Abbreviations: PAAm, polymerized polyacrylamide; BFE, Bessel-fit error; IQR, interquartile range. | | | | | | | | | | |

## **Supplementary Note 2: Histological heterogeneity of all CRLM samples**

Histological evaluation demonstrated considerable intratumoral heterogeneity, including varying fractions of vital tumor cells, necrosis, fibrosis and stroma across all colorectal liver metastasis (CRLM) samples. These features were quantitatively assessed by a board-certified pathologist at our institution. Notably, the percentages reported in **Table S3** differ from the tumor cell fractions underlying the Rubbia-Brandt tumor regression grade (TRG),^[6]^ as the two approaches are based on different assessment strategies. In the original TRG-based evaluation, tumor viability was estimated using predefined categorical intervals (e.g., 5–10%) to stratify treatment response.^[7]^ In contrast, the present analysis provides a continuous and comprehensive quantification of the full tissue composition, including all major components. As a result, the percentage of vital tumor cells reported here reflects a detailed estimation relative to the entire tissue specimen and is not directly comparable to the interval-based TRG categorization. This refined quantification enables a more complete characterization of histological heterogeneity. Detailed data of histological heterogeneity for all 34 samples are provided in **Table S3**.

**Table S3.** Quantitative histopathological composition of 34 CRLM samples. Percentages of vital tumor cells, necrosis, and fibrosis/stroma were assessed by a board-certified pathologist. Treatment response to chemotherapy was classified as no, partial, or major according to the Rubbia-Brandt classification.^[6]^

| **Patient** | **Treatment Response** | **Vital Tumor Cells [%]** | **Necrosis [%]** | **Fibrosis/Stroma [%]** |
| --- | --- | --- | --- | --- |
| 1 | partial | 10 | 85 | 5 |
| 2 | no | 50 | 5 | 45 |
| 3 | no | 40 | 5 | 55 |
| 4 | no | 60 | 10 | 30 |
| 5 | major | 1 | 94 | 5 |
| 6 | no | 60 | 10 | 30 |
| 7 | no | 70 | 0 | 30 |
| 8 | no | 80 | 5 | 15 |
| 9 | no | 30 | 10 | 60 |
| 10 | no | 80 | 5 | 15 |
| 11 | no | 60 | 5 | 35 |
| 12 | major | 5 | 5 | 90 |
| 13 | no | 80 | 5 | 15 |
| 14 | partial | 5 | 90 | 5 |
| 15 | no | 40 | 0 | 60 |
| 16 | no | 40 | 30 | 30 |
| 17 | no | 30 | 40 | 30 |
| 18 | no | 80 | 15 | 5 |
| 19 | partial | 1 | 94 | 5 |
| 20 | no | 30 | 60 | 10 |
| 21 | no | 20 | 10 | 70 |
| 22 | no | 40 | 5 | 55 |
| 23 | no | 60 | 20 | 20 |
| 24 | partial | 20 | 30 | 50 |
| 25 | major | 2 | 73 | 25 |
| 26 | no | 50 | 25 | 25 |
| 27 | no | 70 | 10 | 20 |
| 28 | partial | 5 | 90 | 5 |
| 29 | no | 80 | 15 | 5 |
| 30 | major | 0 | 100 | 0 |
| 31 | major | 0 | 100 | 0 |
| 32 | no | 80 | 15 | 5 |
| 33 | partial | 20 | 50 | 30 |
| 34 | no | 80 | 10 | 10 |

To evaluate whether ttMRE captures this heterogeneity, we analyzed both bulk mechanical parameters and mechanical heterogeneity metrics (**Table S4**). While bulk mechanical parameters shear wave speed *c* and penetration rate *a* did not show significant correlations with histological features, Shannon entropy-based mechanical heterogeneity metrics revealed significant associations. Specifically, Shannon entropy of *c* and joint entropy of *c* and *a* maps showed significant positive correlations with histology-confirmed necrosis (*p* = 0.004 and *p* = 0.016, respectively). In addition, **Figure S3** demonstrates that CRLM samples from major responders exhibited higher necrotic fractions compared to non-major responders (*p* = 0.0005). These findings suggest that ttMRE can capture tumor mechanical heterogeneity on a micro length scale rather than merely reflecting average mechanical properties. In particular, necrotic regions appear to contribute substantially to the observed heterogeneity within the lesions.

**Table S4.** Pearson correlation analysis between biomechanical parameters derived from ttMRE and histopathological features. Bulk parameters include shear wave speed c and penetration rate a, while heterogeneity metrics were quantified using Shannon entropy. Statistically significant correlations (p < 0.05) are indicated in bold.

| **ttMRE parameter** | **Histological feature** | **Pearson correlation coefficient** | ***p* value** |
| --- | --- | --- | --- |
| Bulk *c* [m/s] | Vital Tumor Cells [%] | -0.210 | 0.233 |
|  | Necrosis [%] | 0.246 | 0.161 |
|  | Fibrosis/Stroma [%] | -0.110 | 0.535 |
| Bulk *a* [m/s] | Vital Tumor Cells [%] | -0.270 | 0.122 |
|  | Necrosis [%] | 0.259 | 0.140 |
|  | Fibrosis/Stroma [%] | -0.052 | 0.771 |
| Shannon entropy of *c* [bits] | Vital Tumor Cells [%] | **-0.364** | **0.035** |
|  | Necrosis [%] | **0.482** | **0.004** |
|  | Fibrosis/Stroma [%] | -0.278 | 0.111 |
| Shannon entropy of *a* [bits] | Vital Tumor Cells [%] | -0.336 | 0.052 |
|  | Necrosis [%] | 0.269 | 0.124 |
|  | Fibrosis/Stroma [%] | 0.017 | 0.925 |
| Joint Shannon entropy of *c* and *a* [bits] | Vital Tumor Cells [%] | -0.389 | 0.023 |
|  | Necrosis [%] | **0.410** | **0.016** |
|  | Fibrosis/Stroma [%] | -0.135 | 0.448 |

**Figure S3.** Distribution of necrotic tissue fraction across CRLM samples stratified by treatment response group (no, partial, and major response). Samples from major responders exhibited a higher proportion of necrosis compared to non-major responders, indicating treatment-associated histopathological changes. Asterisks indicate significance levels: *p < 0.05, ***p < 0.001.

## **Supplementary Figure 1: Histograms of pixel-wise shear wave speed *c* values per CRLM sample across treatment response groups**

**Figure S4.** Histograms of shear wave speed c values per CRLM sample across treatment response groups. The distributions of major responders show a broader range of c values compared to the none and partial response groups; the distributions of the none responders in particular have a relative less variation.

## **Supplementary Figure 2: Histograms of pixel-wise penetration rate *a* values per CRLM sample across treatment response groups**

**Figure S5.** Histograms of pixel-wise penetration rate a values per CRLM sample across treatment response groups. Major responders show a broader range of a values than non-responders, indicating greater variability in tissue viscosity. In contrast, non-responders show a relatively narrow a distribution.

## **Supplementary Figure 3: Joint histograms of pixel-wise shear wave speed *c* and penetration rate *a* values per CRLM sample across treatment response groups**

**Figure S6.** Joint histograms of pixel-wise shear wave speed c and penetration rate a values per CRLM sample across treatment response groups. Compared to the non- and partial responders, major responders exhibit a broader joint distribution, with a greater spread of both c and a values, indicating greater biomechanical variability and distributional uncertainty in the CRLM tissue of major responders.

## **Supplementary Note 3: Heterogeneous elastic agar-based phantom preparation**

To test the performance of the spatially resolved pipeline for tabletop magnetic resonance elastography (ttMRE) in heterogeneous environments with elasticity contrast, we prepared an agar-based phantom containing inclusions of varying stiffness. The materials used included agar-agar powder (Merck KGaA, Darmstadt, Germany), distilled water, commercial ultrasound gel (Medimex GmbH, Limburg, Germany), a 3D-printed mold designed to form three cylindrical inclusions, a sealing ring, a syringe with a needle, and glass tubes with plug accessories.

First, a 1.8 wt% agar solution was prepared by dissolving agar powder in heated water and ensuring thorough mixing (250 rpm, 5 minutes, 95°C). The 3D-printed mold with a sealing ring was inserted vertically into the glass tube. The 1.8 wt% agar solution was carefully poured along the inner wall of the tube to avoid bubble formation and to ensure even distribution. After allowing the gel to solidify in a refrigerator for 30 minutes, the mold was gently removed, leaving three cylindrical voids for the inclusions. Next, three materials with distinct mechanical properties were prepared for the inclusions. A 1.2 wt% agar solution was injected into the first void using a syringe and allowed to solidify. A 0.5 wt% agar solution was similarly injected into the second void. Finally, the commercial ultrasound gel was injected into the third void. Once all three voids were filled and the materials solidified, the phantom was sealed using the glass tube's accessories to prevent dehydration.

To provide reference measurements for each material, the remaining 1.8 wt% agar, 1.2 wt% agar, 0.5 wt% agar solution, and ultrasound gel were each used to prepare separate homogeneous phantoms in individual glass tubes, following the same casting and sealing procedures.

## **Supplementary Note 4: Heterogeneous viscoelastic gelatin-based phantom preparation**

To further investigate the performance of the spatially resolved pipeline, we prepared a heterogeneous, viscoelastic gelatin phantom. The phantom comprised two cylindrical inclusions (10 wt% and 35 wt% gelatin) embedded in a homogeneous 25 wt% gelatin matrix. The materials used included gelatin powder (Sigma-Aldrich, St. Louis, USA), distilled water, a copper wire with a 2 mm diameter, a syringe with a needle, and glass tubes with plug accessories.

Due to the stickier nature of gelatin compared to agar, preparation was different. First, a 25 wt% gelatin solution was prepared by hydrating the powder in distilled water and heating to 60 °C with continuous stirring (100 rpm, 15 minutes) to ensure complete dissolution. This solution was poured into a cylindrical glass tube and refrigerated for 1 hour until fully solidified. Next, two cylindrical voids of around 2 mm diameter were created along the z-axis of the tube using the heated copper wire, which was carefully inserted into the gel to locally melt holes for the inclusions. Two additional gelatin solutions (10 wt% and 35 wt%) were then prepared using the same method but kept just above their melting point (~40 °C) to remain injectable and to minimize mixing during filling. These solutions were then carefully injected into the preformed voids using a syringe with a needle. One inclusion was filled with 10 wt% gelatin and the other with 35 wt% gelatin. Once all materials were fully solidified, the phantom was sealed using the glass tube’s accessories.

To obtain reference measurements, homogeneous samples for each gelatin concentration (10 wt%, 25 wt%, and 35 wt%) were prepared using the remaining solutions and cast into separate glass tubes.

## **Supplementary Note 5: Frequency selection and wave attenuation-based exclusion criterion**

The selection of excitation frequencies was guided by constraints on spatial resolution and wave propagation. The upper frequency limit was determined by the spatial resolution of the imaging setup, which defines the shortest wavelength that can be reliably resolved. Conversely, the lower frequency limit was constrained by the sample dimensions to ensure that the wavelength does not exceed the sample size.^[8]^

For the spatially resolved postprocessing pipeline, an additional wave attenuation-based exclusion criterion was applied to ensure sufficient wave propagation within the sample. This criterion was based on the exponential decay of shear wave amplitude in viscoelastic media. Specifically, the retained wave amplitude fraction at the center of the cylindrical sample was estimated as:

$$A\left( R \right)=e^{-k^{''}R} ,$$

(S5)

where $k^{''}$ is the imaginary part of the complex wavenumber ${k^{*}=k^{'}+ik}^{''}$obtained from Bessel fitting, and $R$ is the sample radius. Frequencies were excluded if the retained amplitude fell below a predefined threshold of 0.2%, indicating excessive attenuation and insufficient signal for reliable spatial reconstruction.

## **Supplementary Table 2: ttMRE acquisition parameters for phantom experiments**

**Table S5.** ttMRE acquisition parameters for the homogeneous viscoelastic PAAm phantom, the heterogeneous elastic agar-based and the heterogeneous viscoelastic gelatin-based phantoms as well as their corresponding homogeneous reference phantoms. TE denotes the echo time, TR: the repetition time, T_MEGs_: the duration of MEGs, and G_MEG_: the amplitude of the MEG.

| **Phantom** | | | **ttMRE Parameter** | | | | |
| --- | --- | --- | --- | --- | --- | --- | --- |
|  |  |  | **TE [ms]** | **TR [ms]** | **T_MEGs_ [ms]** | **G_MEG_ [T/m]** | **Vibration Frequency [Hz]** |
| Homogeneous viscoelastic PAAm | | | 28 | 1100 | 0.02 | 0.2 | 500 – 3000 Hz (250 Hz step) |
| Elastic agar-based | Heterogeneous | | 28 | 1950 | 0.02 | 0.3 | 2000 – 2225 Hz (25 Hz step) |
|  | Homogeneous reference | Ultrasound gel | 28 | 3640 | 0.02 | 0.4 | 400 – 2000 Hz (200 Hz step) |
|  |  | 0.5 wt% agar gel | 18 | 2980 | 0.01 | 0.4 | 600 – 2400 Hz (200 Hz step) |
|  |  | 1.2 wt% agar gel | 18 | 2374 | 0.01 | 0.4 | 1700 – 2400 Hz (200 Hz step) |
|  |  | 1.8 wt% agar gel | 18 | 2080 | 0.01 | 0.4 | 1700 – 2400 Hz (200 Hz step) |
| Viscoelastic gelatin-based | Heterogeneous | | 28 | 850 | 0.02 | 0.3 | 3100 – 3400 Hz (100 Hz step) |
|  | Homogeneous reference | 10 wt% gelatin gel | 28 | 1002 | 0.02 | 0.3 | 1400 – 3200 Hz (600 Hz step) |
|  |  | 25 wt% gelatin gel | 28 | 820 | 0.02 | 0.3 | 2600 – 3500 Hz (300 Hz step) |
|  |  | 35 wt% gelatin gel | 28 | 650 | 0.02 | 0.3 | 3000 – 3900 Hz (300 Hz step) |

## **Supplementary Note 6: Bessel-fit error**

In this study, we used the Bessel-fit error (BFE) of the wave field to quantify the deviation of each wave field determined for a sample from the assumed azimuthal symmetry of the homogeneous wave field. This metric guided the selection of an appropriate radial bandpass filter type for the spatially resolved postprocessing pipeline of ttMRE data.

According to Braun *et al.*,^[8]^ all data points of the Cartesian deflection from the wave field were mapped onto cylindrical coordinates and averaged over the azimuthal angle, yielding a single wave profile along radial coordinate *r*. The resulting complex-valued profile, $\overline{\text{u}_{\text{z}}^{\text{*}}}$ , was fitted by the analytical solution of a shear wave in a z-infinite cylinder:

$$\overline{u_{z}^{*}}=A\frac{J_{0}(k^{*}r)}{J_{0}(k^{*}R)}e^{-i\Phi}+O^{*} ,$$

(S6)

where *A* is the wave amplitude, *J_o_* is the Bessel function of the first kind, $k^{*}=k^{'}+ik^{''}$ is the complex wavenumber, *r* is the distance from the data point to the sample origin, *R* is the radius of the sample, *Φ* is the phase of the wave at a radius of the sample *r = R*, *O^*^* is the complex-valued offset term to account for the influence of the compression wave. Residual $\text{r}_{\text{z}}^{\text{*}}$ is the difference between real wave profile $\text{u}_{\text{z}}^{\text{*}}$ and fitted profile $\overline{\text{u}_{\text{z}}^{\text{*}}}$. Finally, the BFE *ϵ* is calculated as:

$$\epsilon=\sqrt{\frac{1}{2N}\sum_{i=1}^{N} \left[ \left( Re(r_{z}^{*}) \right)^{2}+\left( Im(r_{z}^{*}) \right)^{2} \right]} ,$$

(S7)

where *N* is the number of the data points on the wave field.

## **Supplementary References**

[1]	B. Jóźwiak, M. Orczykowska, M. Dziubiński, *PLOS ONE* **2015**, *10*, e0143090.

[2]	A. S. Morr, H. Herthum, F. Schrank, S. Görner, M. S. Anders, M. Lerchbaumer, H.-P. Müller, T. Fischer, K.-V. Jenderka, H. H. G. Hansen, P. A. Janmey, J. Braun, I. Sack, H. Tzschätzsch, *Investigative Radiology* **2022**, *57*, 502.

[3]	L. Martikainen, K. Bertula, M. Turunen, O. Ikkala, *Macromolecules* **2020**, *53*, 9983.

[4]	H. Suzuki, S. Tadano, M. Goto, S. Yamada, K. Fujisaki, I. Kajiwara, M. Suga, G. Nakamura, *Mechanical Engineering Journal* **2015**, *2*, 14.

[5]	L. G. Fonkwe, G. Narsimhan, A. S. Cha, *Food Hydrocolloids* **2003**, *17*, 871.

[6]	L. Rubbia-Brandt, E. Giostra, C. Brezault, A. D. Roth, A. Andres, V. Audard, P. Sartoretti, B. Dousset, P. E. Majno, O. Soubrane, S. Chaussade, G. Mentha, B. Terris, *Annals of Oncology* **2007**, *18*, 299.

[7]	L.-M. Skrip, S. Moosburner, P. Tang, J. Guo, S. Görner, H. Tzschätzsch, K. Brüggemann, K. A. Walter, C. Hosse, U. Fehrenbach, A. Arnold, D. Modest, F. Krenzien, W. Schöning, T. Malinka, J. Pratschke, B. Papke, J. A. Käs, I. Sack, I. M. Sauer, K. H. Hillebrandt, *Journal of Translational Medicine* **2024**, *22*, 774.

[8]	J. Braun, H. Tzschätzsch, C. Körting, A. Ariza De Schellenberger, M. Jenderka, T. Drießle, M. Ledwig, I. Sack, *Magnetic Resonance in Med* **2018**, *79*, 470.
